# Spleen Tyrosine Kinase in Malignancies and Sepsis: Pathophysiological Roles and Biomarker Potential—A Narrative Review

**DOI:** 10.3390/biom16101529

**Published:** 2026-10-20

**Authors:** George Țocu, Bogdan Ioan Ștefănescu, Alina Pleșea Condratovici, Valerii Luțenco, Loredana Stavăr Matei, Lavinia Țocu, Raul Mihailov, Oana Mariana Mihailov, Oana Maria Dragostin, Dana Tutunaru, Ana Maria Pelin, Laurențiu Tony Hangan, Sergiu Ioachim Chirilă, Aysegul Cort, Mihaela Cezarina Mehedinți, Elena Mocanu

**Affiliations:** 1 Research Center in the Medical-Pharmaceutical Field, Faculty of Medicine and Pharmacy, “Dunărea de Jos” University of Galati, 800008 Galati, Romania; george.tocu@ugal.ro (G.Ț.); alina.plesea@ugal.ro (A.P.C.); valerii.lutenco@ugal.ro (V.L.); lavinia.tocu@ugal.ro (L.Ț.); raul.mihailov@ugal.ro (R.M.); oana.mihailov@ugal.ro (O.M.M.); oana.dragostin@ugal.ro (O.M.D.); dana.tutunaru@ugal.ro (D.T.); ana.pelin@ugal.ro (A.M.P.); mihaela.mehedinti@ugal.ro (M.C.M.); 2 “Sf. Apostol Andrei” County Emergency Clinical Hospital, 800578 Galati, Romania; 3 “Sf. Ioan” Children’s Emergency Hospital, 800487 Galati, Romania; 4 “Sfântul Spiridon” Clinical Hospital of Pneumophthisiology, 800552 Galati, Romania; 5 Faculty of Medicine, “Ovidius” University of Constanta, 900470 Constanta, Romania; tony@medcon.ro (L.T.H.); sergiu.chirila@univ-ovidius.ro (S.I.C.); elena.mocanu@univ-ovidius.ro (E.M.); 6 Department of Medical Biochemistry, Faculty of Medicine, Pamukkale University, 20070 Denizli, Turkey; aysegulcort@pau.edu.tr

**Keywords:** spleen tyrosine kinase, malignancies, sepsis, biomarkers, targeted therapy

## Abstract

Spleen tyrosine kinase is a non-receptor tyrosine kinase that couples receptor engagement to cellular survival, immune responses, and tissue injury. This narrative review compares its biology in malignancies and sepsis to examine how cellular compartment, receptor architecture, activation state, and signaling duration alter the meaning of a common signaling hub. A structured narrative search of PubMed/MEDLINE, Scopus, and Web of Science covered English-language publications from January 2000 to August 2026, supplemented by older seminal studies. In several hematological malignancies, sustained receptor signaling or oncogenic cooperation supports malignant-cell persistence; solid tumors show both tumor-promoting and tumor-suppressive functions. In sepsis, dynamically changing immune and vascular responses link spleen tyrosine kinase to pathogen recognition, inflammatory and oxidative responses, endothelial dysfunction, and organ injury. Oncology includes mechanistic studies, human cohorts, and early clinical therapeutic trials, whereas sepsis evidence remains predominantly experimental, with limited human observational and ex vivo validation. Transcript abundance, total protein, and phosphorylation are distinct biomarker endpoints whose interpretation requires identification of the cellular source and sampling context. Kinase inhibitors have shown clinical activity in selected malignancies and protection in experimental sepsis, but these findings do not establish a validated predictive biomarker or clinical efficacy in sepsis. Translation requires disease-specific validation, standardized cellular assessment, and attention to treatment timing and preservation of host defense.

## 1. Introduction

Spleen tyrosine kinase (SYK) is a cytoplasmic non-receptor protein tyrosine kinase that functions as a central signaling node in hematopoietic and immune cells. Structurally, SYK contains two tandem Src homology 2 (SH2) domains connected to a C-terminal catalytic kinase domain through interdomain regions that regulate receptor binding, conformational activation, and tyrosine phosphorylation [1,2]. Although initially characterized as a critical component of B-cell receptor (BCR) signaling, SYK is now recognized as a broader regulator of adaptive and innate immune responses, with functions extending to myeloid-cell activation, platelet signaling, cellular adhesion, phagocytosis, oxidative responses, and vascular biology [2]. Its predominant expression in hematopoietic lineages and its position immediately downstream of several immune receptors make SYK particularly relevant to diseases characterized by altered immune-cell activation [1,2].

SYK is recruited to phosphorylated immunoreceptor tyrosine-based activation motifs (ITAMs) or related motifs present within receptor chains or associated adaptor proteins. This mechanism links SYK to the BCR, fragment crystallizable (Fc) receptors, C-type lectin receptors (CLRs), and several integrin-associated signaling systems [2,3]. Following activation, SYK can engage multiple downstream pathways, including phosphoinositide 3-kinase (PI3K)–protein kinase B (AKT), mitogen-activated protein kinase (MAPK), phospholipase C gamma (PLCγ), and nuclear factor kappa B (NF-κB) signaling, while also influencing calcium mobilization, reactive oxygen species (ROS) generation, phagocytic responses, and inflammasome activity [1,2,3].

The oncological relevance of SYK is particularly well established in hematological malignancies. In B-cell neoplasms, abnormal or sustained BCR–SYK signaling may support malignant-cell survival, proliferation, metabolic activity, and interactions with the tumor microenvironment (TME) [4]. In solid tumors, SYK can promote tumor growth and immunosuppressive signaling in some settings, whereas reduced expression or altered regulation has been associated with loss of tumor-suppressive functions in others [5,6]. These opposing effects make oncology an informative setting in which to examine how lineage, molecular form, and cellular compartment determine the output of SYK signaling.

Sepsis is a syndrome of life-threatening organ dysfunction arising from a dysregulated host response to infection [7]. Its immune and vascular abnormalities provide a contrasting setting for studying SYK: receptor engagement is driven by infectious and host-derived stimuli, and signaling evolves within changing leukocyte and endothelial populations. Murine studies implicate SYK-related mechanisms in renal oxidative injury, endothelial barrier disruption, and cardiac dysfunction [8,9,10]. A nonhuman primate study and a human observational study further associate neutrophil SYK abundance with organ-dysfunction or severity measures [11,12]. These findings concern different experimental systems and analytical endpoints; the expression associations do not themselves demonstrate increased kinase activity.

The scientific value of examining malignancies and sepsis together is therefore comparative. BCR-dependent malignant cells can maintain tonic or chronic active signaling, whereas infected hosts engage Fc receptors, CLRs, integrins, and cooperating innate receptor systems across multiple cellular compartments [2,3,4]. Both contexts recruit overlapping downstream networks, but a persistent survival signal in a malignant clone differs from a time-dependent antimicrobial or inflammatory response in a leukocyte. Solid-tumor cells and tumor-associated immune cells provide an additional test of this distinction because their SYK-dependent effects can oppose one another [5,6]. Comparing these settings asks which properties belong to the kinase and which arise from its receptor, cellular, and temporal environment.

This review follows that question from receptor coupling and downstream mechanisms to measurement and therapeutic interpretation. Expression, phosphorylation, localization, and cellular source are considered separately: a measure of malignant-cell pathway dependence, an immune-population abundance marker, and an acute signaling readout need not have the same biomarker meaning [4,5,6,11,12]. The evidence is also asymmetric. Oncology has a broader mechanistic and translational foundation, with clinical therapeutic investigation in selected malignancies, while direct human validation in sepsis is comparatively limited. Section 4 and Section 5 retain the disease-specific evidence; Section 6 integrates their shared machinery and divergent outputs, providing the basis for the laboratory and therapeutic discussions.

## 2. Materials and Methods

This article was designed as a narrative review of the biological and clinical relevance of spleen tyrosine kinase (SYK) in malignancies and sepsis, with particular emphasis on molecular signaling, disease-specific and shared pathophysiological mechanisms, biomarker assessment, and therapeutic targeting. Relevant literature was identified through targeted searches of PubMed/MEDLINE, Scopus, and Web of Science for English-language publications from January 2000 to August 2026, supplemented by manual examination of the reference lists of relevant articles. Earlier landmark studies were retained when necessary to describe established aspects of SYK structure, activation, receptor coupling, and downstream signaling.

Searches combined terms related to SYK (“spleen tyrosine kinase” OR “SYK”) with terms covering the principal domains of the review, including malignancy and cancer (“cancer”, “malignancy”, “tumor”, “haematological malignancy”, “leukaemia”, “lymphoma”, “multiple myeloma”, and individual solid tumour types), sepsis (“sepsis”, “septic shock”, “infection”, “inflammation”, and “organ injury”), biomarker assessment (“biomarker”, “expression”, “phosphorylation”, “p-SYK”, “immunohistochemistry”, “flow cytometry”, “phospho-flow”, “transcriptomics”, “proteomics”, and “single-cell”), and therapeutic modulation (“SYK inhibitor”, “fostamatinib”, “entospletinib”, “cerdulatinib”, “mivavotinib”, and “targeted therapy”). Search terms were adapted to the syntax and indexing conventions of each database, and additional targeted searches were performed for individual malignancies, signaling pathways, analytical methods, and SYK-directed therapeutic agents when required to address specific sections of the review.

Original experimental and mechanistic studies, translational investigations, human observational studies, biomarker studies, and interventional clinical studies were prioritised when they directly evaluated SYK expression, protein abundance, phosphorylation, signaling, functional involvement, or pharmacological modulation in malignancies or sepsis. Genomic, transcriptomic, proteomic, phosphoproteomic, and single-cell studies were considered when they provided SYK-specific information relevant to the scope of the review. Review articles were used primarily to contextualise established concepts and to identify relevant primary studies. Conference abstracts, editorials and commentaries without substantive original evidence, non-peer-reviewed reports, and publications in which SYK was mentioned only incidentally without contributing directly to the biological, biomarker, or therapeutic questions addressed in this review were generally not included.

Evidence was synthesised narratively according to the disease context, study design and experimental model, biological or cellular source, SYK parameter assessed (mRNA expression, total protein abundance, phosphorylation, or functional activity), and nature of the evidence (experimental, translational, observational, or interventional). Particular attention was given to distinguishing SYK expression or abundance from kinase activation, to separating preclinical findings from evidence obtained in patients, and to identifying concordant and discordant findings between malignancies and sepsis. Because this article was designed as a narrative review, no formal risk-of-bias assessment, evidence-grading framework, or quantitative meta-analysis was undertaken.

## 3. Molecular Biology and Signaling of Spleen Tyrosine Kinase

The biological effects of SYK are determined by a tightly regulated interaction between its structural domains, phosphorylation status, receptor recruitment, and downstream signaling partners. These characteristics enable SYK to function as a molecular interface between extracellular receptor engagement and intracellular responses controlling immune-cell activation, adhesion, migration, phagocytosis, oxidative activity, proliferation, and survival [1,2].

### 3.1. Structure, Expression, Isoforms, and Mechanisms of SYK Activation

The human SYK gene is located on chromosome 9q22 and encodes a cytoplasmic non-receptor tyrosine kinase of approximately 72 kDa. The full-length protein consists of 635 amino acids and contains two tandem N-terminal SH2 domains, a C-terminal catalytic tyrosine kinase domain, and two intervening regulatory regions conventionally referred to as interdomains A and B [13,14,15]. The two SH2 domains form the principal receptor-recognition module and allow SYK to interact with phosphorylated ITAM-containing receptor complexes, whereas interdomain B connects the tandem SH2 region to the catalytic domain and contributes importantly to maintenance of the inactive conformation and regulation of kinase activity [1,2].

SYK was initially considered predominantly a hematopoietic-cell kinase. High expression is found in B lymphocytes and several myeloid lineages, including neutrophils, monocytes/macrophages, mast cells, dendritic cells, and platelets, consistent with its central role in immunoreceptor-mediated signaling [1,2]. Expression is not restricted to the hematopoietic compartment, however, and SYK has also been detected in epithelial cells, fibroblasts, hepatocytes, neuronal cells, mammary tissue, and vascular endothelial cells [16]. This broader cellular distribution is relevant to the diverse functions attributed to SYK in tumor biology, vascular responses, and inflammatory tissue injury. Developmental regulation of SYK and ZAP70 also influences B-cell selection; aberrant ZAP70 expression can redirect signaling toward survival in experimental B-cell malignancies [17].

Alternative splicing generates full-length SykL and the shorter SykS isoform, which lacks 23 residues in interdomain B [18,19]. In cervical cancer models, Lin et al. observed different subcellular distributions and opposing effects of isoform-directed knockdown on proliferation, migration and invasion, with PI3K/AKT pathway involvement [18]. These findings illustrate isoform-dependent biology in a defined tumor context and should not be generalized to every tissue. More recent characterization of platelet SYK isoforms demonstrated that human platelets predominantly express SykL, whereas mouse platelets express both SykL and SykS, emphasizing that isoform distribution may also vary between species and cellular systems [19].

**Figure 1:**
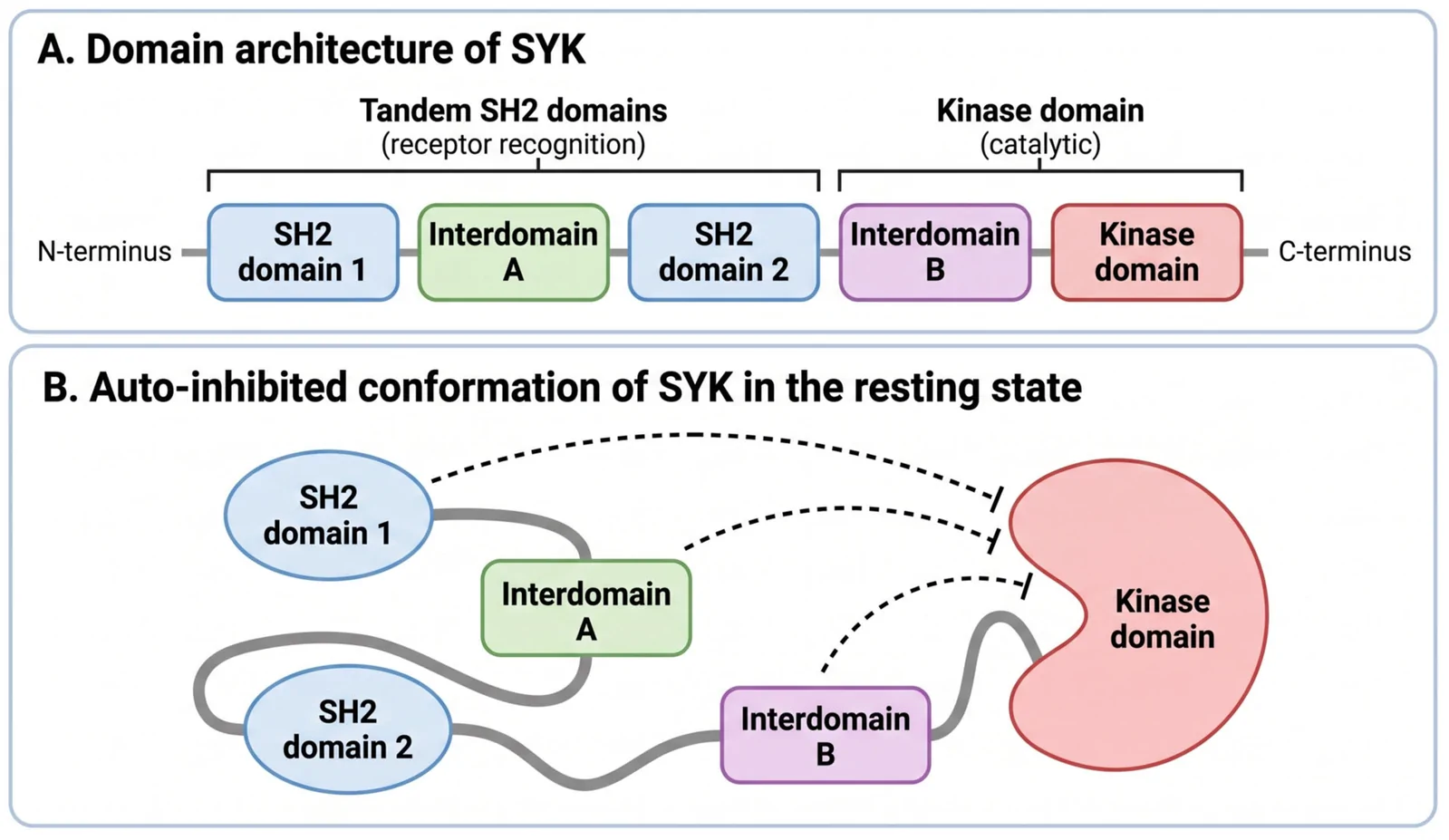
Structural organization and auto-inhibited resting state of spleen tyrosine kinase. (**A**) Domain architecture of spleen tyrosine kinase (SYK), comprising two tandem Src homology 2 (SH2) domains separated by interdomain A, followed by interdomain B and the C-terminal kinase domain. (**B**) Schematic representation of the resting conformation of SYK. Intramolecular interactions involving the SH2-containing regulatory region, interdomain A, interdomain B, and the kinase domain stabilize a closed, auto-inhibited conformation and restrict catalytic activity. Colours distinguish the structural domains: blue indicates the SH2 domains, green interdomain A, purple interdomain B, and red the kinase domain. The continuous gray line represents the polypeptide backbone connecting the domains in their native sequence, whereas dashed lines terminating in inhibitory bars at the kinase domain indicate intramolecular regulatory contacts that stabilize the auto-inhibited conformation and restrict kinase activity. The inhibitory bars are schematic indicators of autoinhibitory regulation, not a map of independently resolved pairwise contacts [2,14,15]. Abbreviations: SH2, Src homology 2; SYK, spleen tyrosine kinase.

In its basal state, SYK adopts an autoinhibited conformation generated by intramolecular interactions between the tandem SH2 regulatory region, interdomain B, and the kinase domain [2,14]. The structural organization of SYK and the intramolecular interactions that stabilize its resting, auto-inhibited conformation are illustrated in Figure 1. Receptor activation promotes phosphorylation of ITAM tyrosine residues, creating high-affinity docking sites for the tandem SH2 domains. Binding to doubly phosphorylated ITAMs induces conformational rearrangements that destabilize the autoinhibitory interaction and increase accessibility of the catalytic domain [14,15]. Experimental phosphoproteomic and biochemical analyses have further shown that ITAM binding can prime SYK for sequential phosphorylation of regulatory tyrosine residues within the SH2–kinase linker, including Tyr352 and Tyr348, followed by phosphorylation events associated with full catalytic activation [14]. Recent structural studies using phospho-ITAM-bound SYK complexes demonstrated that ligand binding restricts SH2-domain mobility and propagates allosteric changes toward the hinge region, thereby disrupting its interaction with the kinase domain and providing a structural basis for receptor-induced SYK activation [15]. SYK can subsequently undergo additional autophosphorylation, generating docking sites for downstream signaling proteins and modulating the magnitude and duration of the cellular response [2,14].

Src-family kinases may initiate receptor and SYK phosphorylation, followed by SYK autophosphorylation and formation of downstream docking sites [2,14]. The human activation-loop residues Tyr525/Tyr526 are frequently used as phosphorylation readouts, but individual phosphosites report different regulatory events and are not interchangeable measures of catalytic output [14]. Receptor recruitment, transphosphorylation, autophosphorylation, and dephosphorylation together determine signaling intensity and duration.

### 3.2. Upstream Receptors and Downstream Signaling Pathways

SYK operates downstream of a broad range of membrane receptors that translate antigen recognition, microbial sensing, immune-complex binding, adhesion, and platelet activation into intracellular signaling responses. The best-characterized upstream systems are ITAM-containing or ITAM-associated receptors, including the BCR, Fc receptors, several CLRs, and platelet immunoreceptors [1,2,3]. Following receptor engagement, Src-family kinases phosphorylate ITAM tyrosine residues, allowing recruitment of SYK through its tandem SH2 domains and subsequent activation of the kinase [2,14]. Some receptors, such as Dectin-1 and C-type lectin-like receptor 2 (CLEC-2), contain a single ITAM-like sequence, generally termed a hemITAM, whereas others, including Dectin-2 and macrophage-inducible C-type lectin (Mincle), signal through ITAM-bearing adaptor proteins such as the Fc receptor gamma chain [2,3]. These receptor systems enable SYK to participate in both adaptive immune signaling and the recognition of microbial or endogenous danger signals.

SYK is also an essential component of integrin-mediated signaling in several hematopoietic cells. In neutrophils, genetic deficiency of SYK markedly impaired β1-, β2-, and β3-integrin-dependent respiratory burst, degranulation, spreading, and actin polymerization, demonstrating that SYK couples adhesion receptors to inflammatory effector functions [20]. In platelets, SYK additionally participates in signaling initiated by glycoprotein VI and CLEC-2, linking immunoreceptor-like pathways to platelet activation and thrombotic responses [2].

Once activated, SYK phosphorylates multiple adaptor and effector proteins that organize downstream signaling complexes. In B cells, an important substrate is B-cell linker protein (BLNK), which provides docking sites for phospholipase C gamma 2 (PLCγ2), Bruton tyrosine kinase (BTK), and additional signaling molecules. Experimental disruption of BLNK markedly impairs SYK-dependent PLCγ2 activation and Rac1–c-Jun N-terminal kinase signaling, establishing BLNK as a major molecular scaffold downstream of SYK [21]. Through these complexes, SYK promotes calcium mobilization and activation of pathways involving PI3K–AKT, MAPK–extracellular signal-regulated kinase (ERK), and NF-κB, thereby regulating proliferation, survival, differentiation, cytoskeletal remodeling, and inflammatory gene expression [1,2].

In innate immune cells, CLR-mediated SYK activation engages a distinct signaling module centered on caspase recruitment domain-containing protein 9 (CARD9). CARD9 associates with B-cell lymphoma/leukemia 10 (BCL10) and mucosa-associated lymphoid tissue lymphoma translocation protein 1 (MALT1), coupling microbial recognition to NF-κB signaling [3]. Brandt et al. identified a phosphorylation switch in CARD9 required for BCL10 engagement and innate inflammatory responses in genetic, biochemical, and myeloid-cell experiments [22]. This pathway is particularly important for responses initiated by Dectin-1 and related receptors during recognition of fungal and other microbial structures [3,22]. Crosstalk between SYK-coupled CLRs and Toll-like receptors (TLRs) can further modify the amplitude and composition of inflammatory responses, indicating that SYK signaling does not operate as an isolated pathway but as part of an integrated pattern-recognition network [3].

SYK signaling also intersects with mechanisms controlling ROS production and inflammasome activation. In experimental models of fungal recognition, SYK was required both for CARD9-dependent synthesis of pro-interleukin-1 beta and for activation of the NOD-like receptor family pyrin domain-containing 3 (NLRP3) inflammasome, with the latter process involving ROS generation and potassium efflux [23]. Experiments with Malassezia species further demonstrate SYK-associated inflammatory cytokine and NLRP3 responses in murine phagocytes [24].

## 4. Spleen Tyrosine Kinase in Malignancies

The malignancy literature offers three complementary settings for examining SYK: receptor-dependent or oncogenic signaling in hematological neoplasms, opposing cell-autonomous functions in solid tumors, and immune modulation within the TME [4,5,6]. Evidence ranges from mechanistic perturbation to human tissue associations and therapeutic trials. The following subsections distinguish these compartments before comparing them with the infection-driven responses in sepsis.

### 4.1. SYK in Hematological Malignancies

Hematological malignancies represent the oncological context in which the pathogenic relevance of SYK has been most consistently demonstrated. In mature B-cell neoplasms, SYK occupies a proximal position within BCR signaling and can sustain survival, proliferation, metabolic adaptation, adhesion, and interactions with the surrounding microenvironment [4]. Unlike kinase-driven malignancies in which recurrent activating mutations directly affect the kinase itself, SYK-dependent transformation often results from persistent receptor signaling, altered pathway regulation, increased kinase expression or phosphorylation, or cooperation with other oncogenic proteins. In selected lymphoid neoplasms, however, chromosomal rearrangements can directly generate constitutively active SYK-containing fusion proteins.

In diffuse large B-cell lymphoma (DLBCL), experimental evidence indicates that SYK-dependent BCR signaling contributes to malignant B-cell survival. Cabaud Gibouin et al. demonstrated that inhibition of heat shock protein 110 (HSP110) reduced the survival of activated B-cell-like DLBCL (ABC-DLBCL) cells and decreased the phosphorylation of key BCR signaling kinases, including SYK and BTK. Mechanistic analyses further identified an interaction between HSP110 and SYK, supporting a regulatory role for HSP110 in SYK phosphorylation. Moreover, combined inhibition of HSP110 and PI3K synergistically reduced SYK, BTK, and AKT phosphorylation and suppressed tumor growth in experimental DLBCL models, including patient-derived xenografts [25].

**Figure 2:**
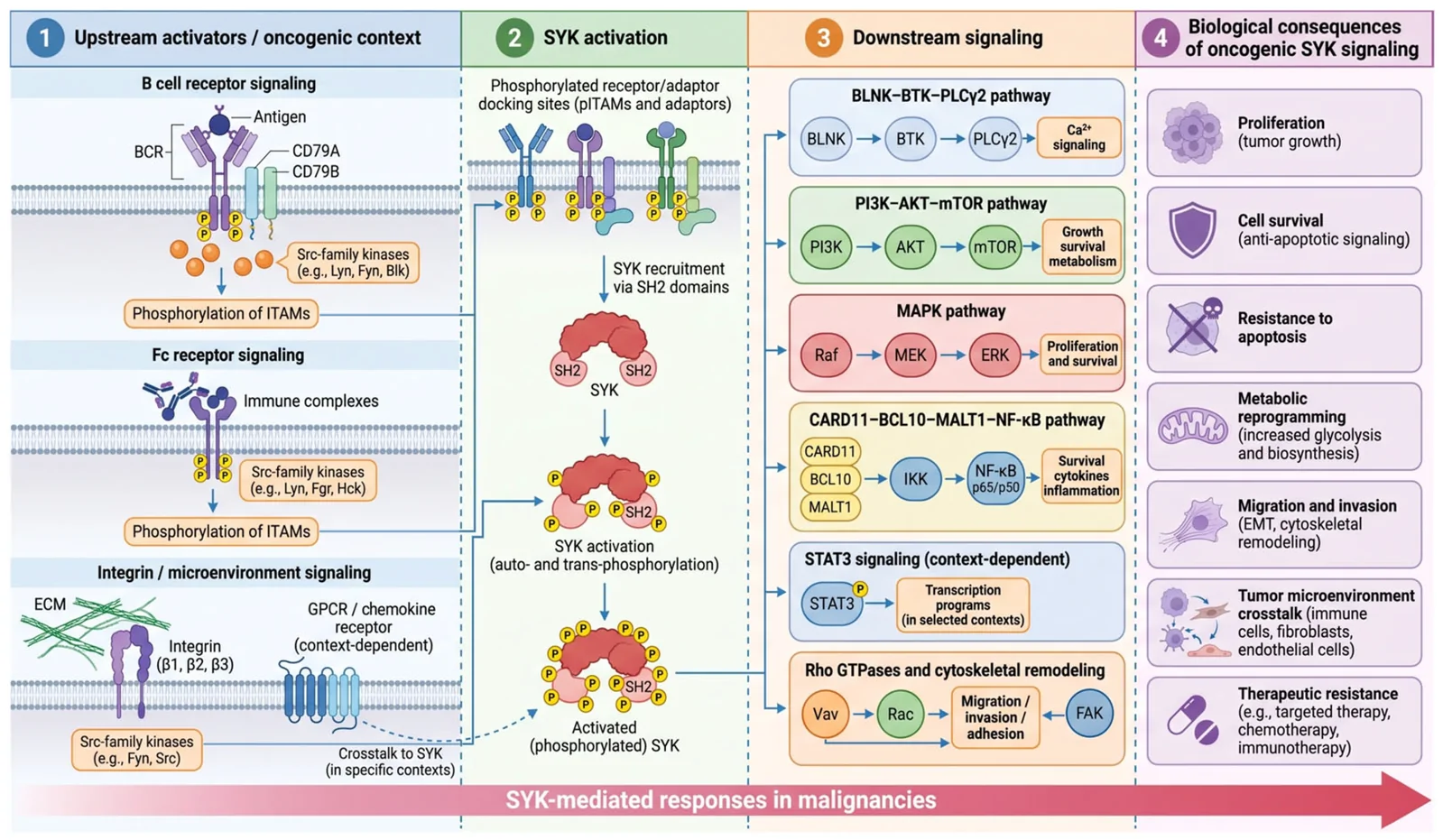
SYK activation and downstream signaling in malignancies. The figure illustrates major oncogenic signaling contexts leading to spleen tyrosine kinase (SYK) recruitment and activation and the principal downstream pathways associated with malignant-cell behavior. B-cell receptor (BCR) and activating Fc gamma receptor (FcγR) signaling promote Src-family kinase-dependent phosphorylation of immunoreceptor tyrosine-based activation motifs (ITAMs), generating phosphorylated receptor/adaptor docking sites that recruit SYK through its tandem Src homology 2 (SH2) domains. Integrin signaling can promote SYK activation through Src-family kinases and distinct ITAM-independent or adaptor-mediated mechanisms. The dashed receptor-crosstalk input represents selected chemokine and microenvironmental interactions [29]. Activated and phosphorylated SYK engages multiple downstream signaling pathways, including BLNK–BTK–PLCγ2-mediated Ca^2+^ signaling, PI3K–AKT–mTOR, MAPK, CARD11–BCL10–MALT1–NF-κB, and cytoskeletal remodeling involving the Vav–Rac axis. Focal adhesion kinase (FAK) is depicted as a related integrin-associated regulator of adhesion and motility that may functionally converge with SYK-dependent cytoskeletal signaling, rather than as an established direct SYK phosphorylation target [6,29]. The STAT3 module denotes context-dependent STAT3-associated signaling downstream of SYK [30]. These interconnected pathways can promote proliferation, cell survival, resistance to apoptosis, metabolic reprogramming, migration and invasion, tumor–microenvironment crosstalk, and therapeutic resistance. The figure specifically summarizes oncogenic SYK signaling and does not depict the tumor-suppressive functions of SYK described in selected solid tumors. The modules summarize selected cellular contexts rather than a universal sequence of direct kinase–substrate reactions. Fc-receptor input may also reflect tumor-associated immune cells, and the STAT3 and microenvironmental connections are context-dependent [2,25,26,27,30,31,32,33]. Colours are used to distinguish the main signalling modules and biological consequences for visual clarity and do not indicate a universal molecular classification or signalling hierarchy. Abbreviations: AKT, protein kinase B; BCL10, B-cell lymphoma/leukemia 10; BCR, B-cell receptor; BLNK, B-cell linker protein; BTK, Bruton tyrosine kinase; CARD11, caspase recruitment domain family member 11; CD, cluster of differentiation; ECM, extracellular matrix; EMT, epithelial–mesenchymal transition; ERK, extracellular signal-regulated kinase; FAK, focal adhesion kinase; FcγR, Fc gamma receptor; GPCR, G protein-coupled receptor; IKK, IκB kinase; ITAM, immunoreceptor tyrosine-based activation motif; MALT1, mucosa-associated lymphoid tissue lymphoma translocation protein 1; MAPK, mitogen-activated protein kinase; MEK, mitogen-activated protein kinase kinase; mTOR, mechanistic target of rapamycin; NF-κB, nuclear factor kappa B; P, phosphorylation; PI3K, phosphoinositide 3-kinase; pITAM, phosphorylated immunoreceptor tyrosine-based activation motif; PLCγ2, phospholipase C gamma 2; Rac, Ras-related C3 botulinum toxin substrate; SH2, Src homology 2; STAT3, signal transducer and activator of transcription 3; SYK, spleen tyrosine kinase.

Subsequent work distinguished different forms of BCR dependence across molecular DLBCL subtypes. In activated B-cell-like (ABC) DLBCL, chronic active BCR signaling is maintained through receptor clustering and recurrent alterations involving CD79A or CD79B, thereby sustaining downstream survival pathways [26]. By contrast, germinal center B-cell-like (GCB) DLBCL can depend predominantly on tonic BCR signaling, in which SYK and CD19 contribute to AKT activation even in the absence of antigen-driven receptor clustering [27]. These observations illustrate that SYK dependence cannot be interpreted uniformly across DLBCL, because the biological consequences of its activation are determined by the architecture and intensity of the upstream BCR signal. In Myd88-driven mouse lymphoma models, an inducible Cd79b mutation conferred sensitivity to ibrutinib, adding genotype-specific evidence of BCR-pathway dependence [28]. The principal receptor-dependent and oncogenic SYK signaling pathways, together with related integrin-associated mechanisms involved in cellular adhesion and migration, are schematically summarized in Figure 2.

SYK-directed pharmacodynamic evidence is also available in CLL. In a correlative investigation involving 17 patients with relapsed/refractory CLL, Lam et al. observed reductions in STAT3 phosphorylation and MCL1 protein abundance after entospletinib treatment; subsequent entospletinib–obinutuzumab therapy was associated with changes in T-cell phenotype and cytokine secretion [34]. These treatment-associated findings support investigation of pathway modulation but do not establish baseline SYK overexpression or genetic dependence. Importantly, SYK activity in CLL extends beyond classical BCR signaling. Chemokine and integrin stimulation can induce SYK phosphorylation and downstream AKT activation, enhancing migration toward CXCL12, adhesion to stromal ligands, and protection from drug-induced apoptosis [29]. These findings indicate that SYK contributes to the bidirectional communication between malignant B cells and supportive stromal niches, thereby linking receptor signaling to trafficking, tissue retention, and therapeutic resistance. This microenvironment-dependent component is particularly relevant because CLL cell behavior is strongly influenced by signals encountered in lymphoid tissues and bone marrow rather than by autonomous proliferation alone.

A distinct role for SYK has been identified in acute myeloid leukemia (AML). Polak et al. found that R406 reduced oxidative metabolism, clonogenic potential, and leukemia stem-cell-associated populations in AML models; ex vivo treatment of primary blasts also reduced subsequent engraftment in immunodeficient mice [35]. These pharmacological and metabolic findings complement the mechanistic evidence for SYK in AML. Subsequent mechanistic work identified direct binding and transactivation of FMS-like tyrosine kinase 3 (FLT3), particularly in AML carrying an internal tandem duplication (FLT3-ITD). SYK phosphorylation at Tyr525/Tyr526, oncogenic cooperation, MYC-associated transcriptional programs, and resistance to FLT3-directed therapy were examined in experimental models and primary AML material [36]. Targeting the lipid-binding function of SYK has also shown preclinical activity in AML, extending therapeutic investigation beyond ATP-site inhibition [37]. Boros et al. then separated two relevant observations: higher baseline phosphorylated SYK (p-SYK) at Tyr323 and Tyr525/Tyr526 correlated with inhibitor sensitivity in AML cell lines, while p-SYK Tyr323 immunohistochemistry in 70 primary bone marrow biopsies associated with unfavorable outcome [38]. Tyr323 staining was supported as an activity surrogate by comparison with Tyr525/Tyr526 phosphorylation in cell lines; the patient cohort did not validate prediction of clinical response to a SYK inhibitor.

SYK can also participate directly in oncogenic transformation through gene fusion. A recurrent t(5;9)(q33;q22) translocation identified in a subset of peripheral T-cell lymphoma (PTCL) generates an interleukin-2-inducible T-cell kinase (ITK)–SYK fusion containing the N-terminal membrane-targeting region of ITK and the catalytic domain of SYK [39]. Functional studies demonstrated that ITK–SYK localizes constitutively to membrane lipid rafts and reproduces proximal T-cell receptor signaling in an antigen-independent manner. Conditional expression of the fusion protein in mice produced highly penetrant PTCL resembling the human disease, providing direct evidence that constitutive SYK kinase activity can function as an oncogenic driver when coupled to an inappropriate signaling scaffold [40]. A separate model links chronic TCR stimulation to epigenetic acquisition of NK-receptor/SYK signaling in peripheral T-cell lymphoma, indicating a non-fusion route to pathway dependence [41].

The importance of SYK is not restricted to DLBCL, CLL, AML, and PTCL. In mantle cell lymphoma (MCL), genomic and expression analyses have identified SYK amplification or overexpression in selected cases, while functional inhibition can reduce proliferation in SYK-dependent cells [42]. More recently, phospho-specific flow cytometry of primary MCL samples demonstrated marked interpatient heterogeneity in BCR signaling responses; greater SYK responsiveness was associated with inferior overall survival, supporting the concept that pathway activation rather than total protein abundance alone may carry clinically relevant information [43]. Unlike a single resting abundance measurement, the MCL stimulation assay interrogates receptor responsiveness; an analogous distinction between abundance and stimulus-dependent function is relevant when assessing immune cells in sepsis [12,43]. Recent loss-of-function and mechanistic experiments identify CEACAM1 as a facilitator of SYK recruitment and BCR signaling in a subset of mantle cell lymphomas [44].

### 4.2. SYK in Solid Tumors and Its Context-Dependent Functions

In contrast to hematological malignancies, where SYK most frequently supports oncogenic receptor signaling, its function in solid tumors is substantially more heterogeneous. Evidence accumulated across epithelial and non-hematopoietic cancers indicates that SYK can act as either a tumor suppressor or a tumor-promoting kinase depending on tumor lineage, isoform expression, intracellular localization, signaling partners, and the biological state of the malignant cell [5,6]. This functional duality is particularly evident when comparing breast, pancreatic, melanoma, hepatic, ovarian, and colorectal tumor models. A computational comparison of SYK signaling networks also identified potential molecular determinants of these opposing functions, complementing the disease-specific experimental evidence [6].

The tumor-suppressive role of SYK was initially demonstrated in breast cancer. Coopman et al. showed that SYK expression was readily detectable in normal mammary epithelium, benign breast lesions, and breast cancer cells with low tumorigenic potential, whereas its expression was markedly reduced or absent in invasive breast carcinoma cells [45]. Reintroduction of wild-type SYK into SYK-negative breast cancer cells suppressed tumor growth and metastatic dissemination in vivo, whereas expression of a kinase-deficient form enhanced tumor formation [45]. Epigenetic regulation was subsequently identified as one mechanism responsible for loss of SYK expression. Yuan et al. detected promoter CpG hypermethylation in 30% of breast cancer cell lines and 32% of primary breast tumors, with methylation strongly associated with transcriptional silencing; demethylating treatment restored SYK expression in previously negative cells [46]. These findings supported the concept that loss of SYK expression and its associated tumor-suppressive function can facilitate acquisition of an invasive phenotype rather than merely reflecting tumor progression.

A similar tumor-suppressive pattern has been reported in pancreatic ductal adenocarcinoma (PDAC) and melanoma. In PDAC, SYK expression progressively declined with loss of tumor differentiation, and restoration of SYK in deficient pancreatic cancer cells reduced anchorage-independent growth, tumor formation, and cellular invasion [47]. SYK re-expression was accompanied by decreased cyclin D1 and AKT expression and altered regulation of invasion-associated proteins, while promoter methylation contributed to its loss in poorly differentiated cells [47]. In melanoma, epigenetic silencing of SYK was also observed in subsets of malignant cells. Experimental restoration of SYK suppressed clonogenic growth and three-dimensional invasion and induced a senescence-like phenotype involving p53-dependent induction of p21 and reduced proliferative activity [48]. Thus, in these tumor types, preservation of SYK appears to constrain malignant progression through mechanisms involving differentiation, cell-cycle regulation, senescence, adhesion, and invasion.

However, SYK cannot be classified universally as a tumor suppressor in solid malignancies. Hepatocellular carcinoma provides a particularly informative example because the biological effect depends on the expressed SYK isoform. Hong et al. found that the full-length SykL isoform was downregulated in a substantial proportion of hepatocellular carcinomas and exerted anti-invasive and pro-apoptotic effects, whereas the shorter SykS isoform was detected preferentially in tumors and was associated with vascular invasion, multiple tumor lesions, and epithelial–mesenchymal transition (EMT) [49]. Functionally, SykS promoted tumor growth, suppressed apoptosis, and enhanced EMT through ERK signaling, directly opposing several effects of SykL [49]. Patients whose tumors retained SykL without detectable SykS had more favorable survival and recurrence profiles, illustrating that measurement of total SYK alone may obscure biologically opposite effects generated by alternative splice variants.

Tumor-promoting SYK signaling has also been identified in selected breast and ovarian cancer contexts. A systems-level pharmacological and phosphoproteomic analysis of triple-negative breast cancer (TNBC) identified SYK as a functional dependency in a subset of basal-like tumors. SYK inhibition selectively induced apoptosis and suppressed tumor growth, while phosphoproteomic analyses linked this sensitivity to disruption of signal transducer and activator of transcription 3 (STAT3) signaling [30]. These findings differed markedly from the earlier tumor-suppressor model of breast cancer and indicated that molecular state, rather than tissue of origin alone, can determine whether SYK activity supports or restrains malignant-cell fitness [30]. In ovarian cancer models, SYK function promoted migration and invasion through phosphorylation of cytoskeletal regulators, including cortactin and cofilin; SYK Tyr525/Tyr526 phosphorylation was also assessed. Genetic or pharmacological SYK inhibition reduced actin polymerization and impaired invasion in both conventional and three-dimensional tumor models [50].

Recent work has further expanded the tumor-promoting functions of SYK beyond classical growth and migration pathways. In high-grade serous ovarian cancer and TNBC, SYK was shown to participate directly in the DNA damage response by promoting DNA double-strand break resection and homologous recombination repair [51]. This activity increased resistance to DNA-targeted treatments, indicating that SYK expression may influence not only tumor-cell behavior but also therapeutic vulnerability [51]. In colorectal cancer, isoform-specific analyses further illustrate the complexity of SYK biology. Although global SYK depletion may enhance proliferation and motility in some experimental settings, selective suppression of the full-length SYK(L) isoform reduces cell viability and induces apoptosis, suggesting that established tumors may retain an isoform-specific dependency on SYK signaling [52].

The isoform and DNA-repair findings distinguish solid-tumor SYK biology from a simple model of persistent BCR-dependent survival. They also caution against transferring a therapeutic interpretation from one cellular setting to another, including infection, without evidence of the relevant signaling dependency [5,6,49,51,52].

### 4.3. SYK in the Tumor Microenvironment

Beyond its cell-autonomous effects in malignant cells, SYK can influence tumor progression through reciprocal interactions between cancer cells, immune populations, and stromal components within the TME. Its broad expression across myeloid and lymphoid lineages enables SYK-dependent signaling to modify macrophage polarization, lymphocyte recruitment, inflammatory mediator production, and adaptive antitumor immunity.

Human tissue and cellular evidence supports an immunomodulatory function of tumor-cell SYK. Aguirre-Ducler et al. spatially assessed SYK in lung and breast carcinoma cohorts and found associations between compartment-specific expression, T-cell infiltration, and more favorable outcomes [53]. In cultured tumor cells, tumor necrosis factor alpha (TNF-α) increased SYK expression, and TAK-659 modified TNF-α-induced inflammatory signaling and T-cell responses. This compound inhibits both SYK and FLT3; FLT3 was not detected in the tested lung cell lines, providing a relevant cellular specificity control [53]. Paired biopsies from three patients with advanced triple-negative breast cancer showed reduced tumor-infiltrating lymphocytes and T-cell proliferation after TAK-659 exposure following nivolumab [53]. This exploratory observation is consistent with an immune-supportive role for tumor-cell SYK, but the small sample, sequential treatment, and dual-target pharmacology prevent an isolated causal attribution to SYK inhibition in patients.

Conversely, SYK activity within tumor-associated macrophages (TAMs) can promote an immunosuppressive microenvironment. Joshi et al. demonstrated in syngeneic lung carcinoma and melanoma models that a macrophage SYK–PI3K axis supported tumor growth and metastasis by promoting immunosuppressive macrophage polarization [32]. Genetic suppression of macrophage SYK and pharmacological targeting, including a dual SYK–PI3K inhibitor, shifted transcriptional programs toward a proinflammatory phenotype, enhanced cluster of differentiation 8-positive (CD8+) T-cell recruitment and activity, destabilized hypoxia-inducible factor 1 alpha (HIF-1α) under hypoxic conditions, and strengthened antitumor immunity [32]. The dual inhibitor cannot isolate the contribution of SYK alone, whereas the genetic experiments strengthen the macrophage-specific mechanistic interpretation [32].

Rohila et al. identified abundant SYK expression in pancreatic ductal adenocarcinoma-associated macrophages and assessed Tyr348 phosphorylation by flow cytometry and immunoblotting [33]. In isolated murine TAMs, this phosphorylation was induced by Fc gamma receptor ligation and α4β1 integrin engagement, whereas LPS did not produce the same response, illustrating receptor- and compartment-specific coupling. Myeloid-specific SYK deletion reduced tumor growth and metastasis while increasing CD8+ T-cell infiltration, proliferation, and cytotoxic activity [33]. Pharmacological inhibition also reprogrammed macrophages and counteracted gemcitabine-induced immunosuppression in murine tumors and human pancreatic tumor slice cultures. In diffuse glioma, elevated SYK expression associated with macrophage infiltration, immune-checkpoint expression, and unfavorable prognosis; these abundance associations differ from the phosphorylation and genetic evidence in the PDAC models [54].

Compartment-resolved analyses are therefore essential: a bulk tumor signal may combine malignant cells, macrophages, B cells, and other infiltrating populations [32,33,53,54]. A related problem arises in septic blood, where changes in leukocyte composition or low-density neutrophil frequency may alter total SYK measurements without establishing a uniform change in kinase activity [11,12]. The shared analytical challenge is to identify the responding population before assigning biological meaning to the signal.

Representative malignancy studies, including mechanistic experiments, human cohorts, and clinical trials, are summarized by tumor type in Supplementary Table S1.

## 5. Spleen Tyrosine Kinase in Sepsis

In sepsis, SYK is studied within a changing host response involving microbial recognition, leukocyte effector functions, vascular integrity, and organ injury [7]. In contrast to the sustained dependencies identified in selected malignant cells, its outputs here depend on infectious stimulation and the simultaneous antimicrobial and immunoregulatory programs of the responding populations [2,10,11,12]. Most causal and therapeutic findings derive from cellular or murine models. Nonhuman primate observations, human blood studies, and patient-derived ex vivo experiments provide translational support, but should be distinguished from clinical validation of a biomarker or treatment.

### 5.1. Pathogen Recognition and Innate Immune Signaling

The innate immune response to infection begins with recognition of conserved microbial structures by pattern-recognition receptors (PRRs) expressed on or within innate immune cells. SYK participates in this process primarily downstream of receptors containing ITAM or hemITAM signaling motifs, as well as through functional interactions with other PRR families [2,3]. Among the best-characterized SYK-coupled receptors are Fc receptors and several CLRs, including Dectin-1, Dectin-2, and Mincle. Engagement of these receptors promotes recruitment and phosphorylation of SYK, followed by activation of signaling modules that regulate cytokine production, phagocytosis, oxidative responses, and antimicrobial effector functions [3,22].

Dectin-1 provides a well-established model of direct SYK-dependent microbial recognition. Binding of fungal β-glucans induces phosphorylation of the receptor hemITAM and recruitment of SYK, which subsequently activates CARD9-dependent signaling and inflammatory gene expression [3,22]. SYK-dependent signaling can also cooperate with TLR pathways rather than acting independently. Dennehy et al. demonstrated in cellular macrophage experiments that Dectin-1–SYK signaling synergized with several TLRs, including TLR2 and TLR4, and that both SYK and myeloid differentiation primary response 88 (MyD88) were required for optimal collaborative cytokine production [55]. This interaction prolonged degradation of inhibitor of NF-κB (IκB) and enhanced nuclear NF-κB signaling, illustrating how simultaneous recognition of different microbial structures can amplify innate immune responses [55]. Such receptor cooperation is particularly relevant to sepsis, where immune cells are exposed to complex combinations of microbial ligands rather than to a single isolated agonist. Super-resolution imaging in macrophages has further linked Dectin-1–TLR2 cooperation to the spatial organization of receptor and downstream signaling components [56].

The relationship between SYK and TLR signaling involves intracellular adaptor cooperation. Using murine RAW264.7 cells, mouse peritoneal macrophages and complementary biochemical experiments, Yi et al. demonstrated functional cooperation between MyD88 and SYK in inflammatory signaling [57]. SYK loss impaired NF-κB activation, while MyD88 perturbation affected inflammatory effector responses. These cellular findings support an adaptor-dependent mechanism but do not establish direct human monocyte TLR4–SYK association or kinase activation in patients with sepsis. Liu et al. subsequently identified the transmembrane adaptor SLP adaptor and C-terminal Src kinase-interacting membrane protein (SCIMP) as a platform recruiting SYK after LPS-induced TLR4 stimulation in macrophage models; phospho-SYK was assessed with a Tyr525-directed antibody, and SYK contributed to SCIMP and TLR4 phosphorylation and selective cytokine outputs [58]. Thus, receptor cooperation can recruit the shared kinase through adaptor architecture distinct from the BCR scaffold prominent in B-cell malignancies.

A direct link between this receptor crosstalk and experimental sepsis was demonstrated by Voss et al. in murine models of endotoxemia and septic peritonitis. The investigators identified CD300b as an LPS-binding receptor that associates with TLR4 through an ITAM-containing adaptor in macrophages. Formation of this receptor complex promoted recruitment and activation of SYK and PI3K and altered MyD88- and Toll/interleukin-1 receptor domain-containing adapter-inducing interferon beta (TRIF)-dependent signaling, resulting in enhanced inflammatory responses and increased severity of septic shock [59]. Macrophage depletion and adoptive-transfer experiments further identified CD300b-expressing macrophages as an important cellular component of this response [59]. This study is particularly relevant because it demonstrates that SYK-dependent receptor integration can modify the severity of systemic bacterial inflammation in vivo rather than representing only an isolated signaling phenomenon observed in cultured cells.

**Figure 3:**
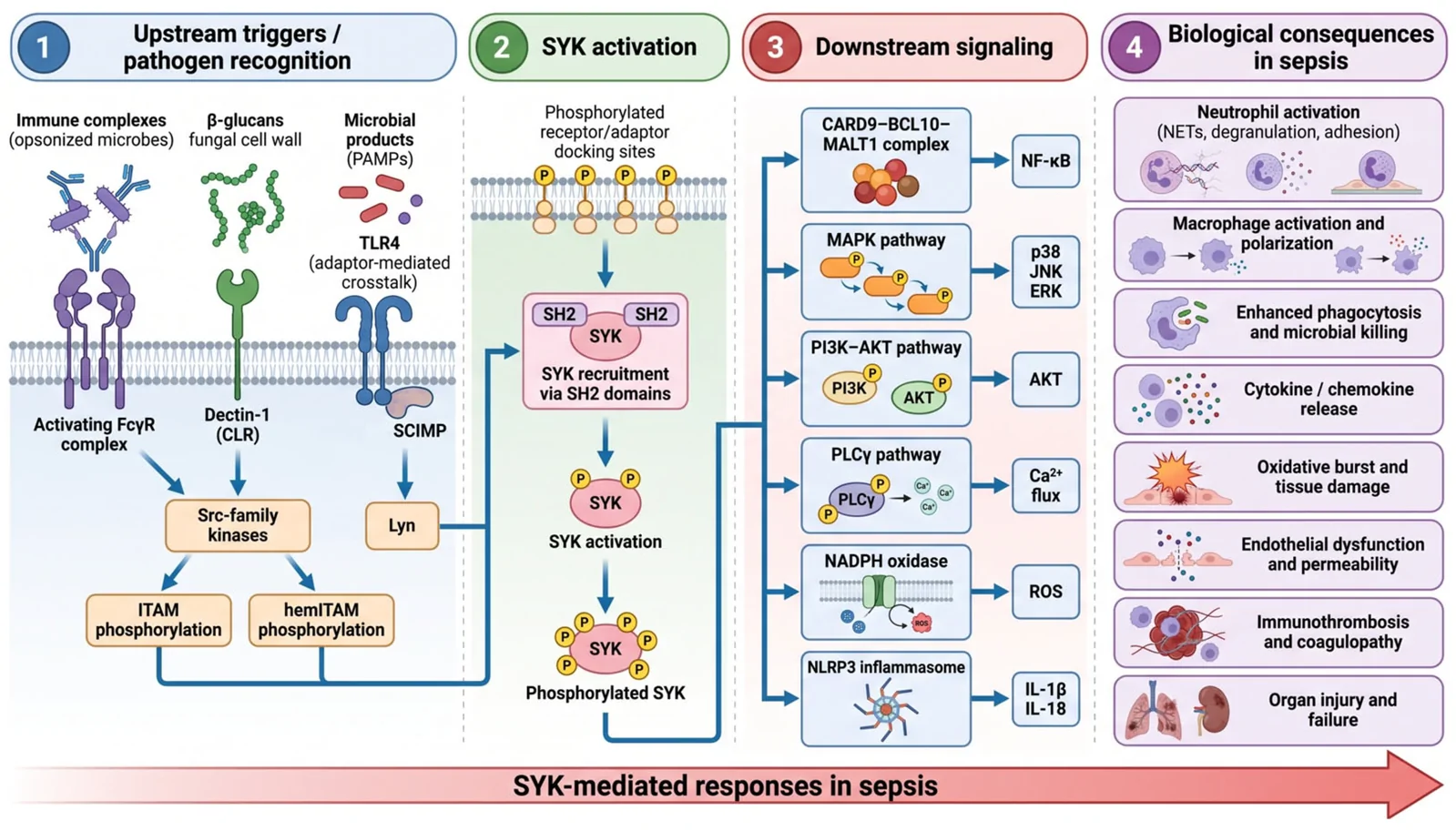
SYK activation and downstream signaling in sepsis. The figure illustrates major receptor- and adaptor-mediated mechanisms leading to SYK activation and their downstream consequences in sepsis. Immune complexes engage activating FcγR complexes, whereas β-glucans activate Dectin-1, leading to Src-family kinase-dependent phosphorylation of ITAM and hemITAM motifs, respectively. In parallel, TLR4–SCIMP signaling engages Lyn-dependent adaptor phosphorylation, generating docking sites that can facilitate SYK recruitment. These phosphorylated receptor or adaptor docking sites enable recruitment of SYK through its tandem SH2 domains, followed by kinase activation and phosphorylation. Activated and phosphorylated SYK engages multiple downstream pathways, including CARD9–BCL10–MALT1/NF-κB, MAPK, PI3K–AKT, PLCγ-mediated Ca^2+^ signaling, NADPH oxidase-dependent ROS generation, and NLRP3 inflammasome activation. The resulting responses include neutrophil and macrophage activation, enhanced phagocytosis and microbial killing, cytokine and chemokine release, oxidative tissue injury, endothelial dysfunction, immunothrombosis, coagulopathy, and organ injury. The relative contribution of individual pathways depends on the receptor involved, immune-cell population, infectious stimulus, and stage of sepsis. This is a mechanistic synthesis of experimental receptor signaling and possible downstream consequences, rather than a map validated in patients with sepsis. NLRP3 coupling includes evidence from fungal-recognition models; clinical endothelial and coagulation abnormalities do not independently prove SYK-specific causality [9,10,23,58,59]. P denotes phosphorylation. Colours are used to distinguish the principal stages of SYK signalling, receptor-associated components, downstream pathways, and biological consequences for visual clarity, without implying a universal molecular classification. Abbreviations: AKT, protein kinase B; BCL10, B-cell lymphoma/leukemia 10; CARD9, caspase recruitment domain-containing protein 9; CLR, C-type lectin receptor; ERK, extracellular signal-regulated kinase; FcγR, Fc gamma receptor; hemITAM, half immunoreceptor tyrosine-based activation motif; IL, interleukin; ITAM, immunoreceptor tyrosine-based activation motif; JNK, c-Jun N-terminal kinase; MALT1, mucosa-associated lymphoid tissue lymphoma translocation protein 1; MAPK, mitogen-activated protein kinase; NADPH, nicotinamide adenine dinucleotide phosphate; NETs, neutrophil extracellular traps; NF-κB, nuclear factor kappa B; NLRP3, NOD-like receptor family pyrin domain-containing 3; PAMPs, pathogen-associated molecular patterns; PI3K, phosphoinositide 3-kinase; PLCγ, phospholipase C gamma; ROS, reactive oxygen species; SCIMP, SLP adaptor and C-terminal Src kinase-interacting membrane protein; SH2, Src homology 2; SYK, spleen tyrosine kinase; TLR4, Toll-like receptor 4.

Downstream of these receptor-proximal events, SYK can connect microbial recognition to CARD9–BCL10–MALT1 and NF-κB signaling, MAPK activation, PI3K–AKT signaling, calcium-dependent responses, ROS generation, and inflammasome activation [3,22,23]. The precise combination of pathways engaged varies according to the microbial ligand, receptor repertoire, and responding immune-cell population. The major upstream signals converging on SYK and the downstream pathways linking its activation to inflammatory and organ-level consequences in sepsis are summarized in Figure 3. These signaling mechanisms provide the molecular framework for the cell-specific neutrophil, monocyte, and macrophage responses discussed in the following section.

### 5.2. Neutrophil, Monocyte, and Macrophage Responses

Neutrophils, monocytes, and macrophages represent major cellular effectors of the innate immune response in sepsis, and SYK participates in several signaling processes that determine their activation, migration, antimicrobial activity, and inflammatory output. In neutrophils, SYK couples Fc receptors, integrins, and other immunoreceptor-associated pathways to cytoskeletal remodeling, degranulation, oxidative responses, and adhesion [2,20]. These functions are essential for early pathogen control but may become detrimental when excessive or prolonged activation promotes endothelial injury, microvascular obstruction, and tissue damage. In mouse acute lung injury, neutrophil-specific Shp1 loss caused severe pulmonary hemorrhage that was rescued by additional Syk deletion, illustrating the consequences of dysregulated neutrophil signaling [60].

In a murine model of sepsis-associated acute kidney injury (AKI), Al-Harbi et al. observed increased total SYK expression in neutrophils and dendritic cells, alongside inflammatory and oxidative abnormalities [8]. Neutrophil findings included increased inducible nitric oxide synthase, nicotinamide adenine dinucleotide phosphate oxidase 2, and nitrotyrosine. R406 reduced these abnormalities, dendritic-cell cytokine production, and renal dysfunction [8]. These measurements concern expression rather than phosphorylation or kinase activation. The inhibitor response supports involvement of a pharmacologically modifiable SYK-related pathway, while exclusive target attribution is less secure without independent genetic confirmation.

Warner et al. examined healthy-donor human neutrophils stimulated ex vivo with LPS from Gram-negative bacteria [61]. R406 reduced neutrophil extracellular trap (NET) formation, degranulation, ROS generation, endothelial adhesion, and emergence of cluster of differentiation 16-low (CD16low) phenotypes. Treated neutrophils retained measured phagocytosis, cytokine release, migration toward interleukin-8, and metabolic activity [61]. These findings concern selected functions under the tested stimulation conditions; they do not establish preservation of antimicrobial immunity during systemic treatment of sepsis. Because the experiments predominantly used R406, the findings are best described as pharmacological evidence consistent with SYK involvement rather than proof that every affected response is exclusively SYK-dependent.

In a translational nonhuman primate model of Klebsiella pneumoniae-induced septic shock, Teague et al. followed six infected animals and found time-dependent increases in total SYK expression in whole-blood neutrophils (WBNs) and low-density neutrophils (LDNs), with SYK-positive subsets showing higher myeloperoxidase (MPO) and lactoferrin expression [11]. SYK-positive LDNs correlated with creatinine, prothrombin time, fibrinogen, and tissue SYK staining. Two animals had been allocated to fostamatinib in a concurrent blinded evaluation; the report did not provide an unblinded treatment-efficacy comparison [11]. The findings support temporal and cellular associations, rather than clinical biomarker or therapeutic validation.

In the subsequent human observational study, 35 patients with sepsis and 18 healthy donors underwent neutrophil phenotyping; clustering used 23 patient samples, and ex vivo stimulation/inhibition used samples from 16 patients [12]. Conventional intracellular flow cytometry measured total SYK, not phospho-SYK. SYK-positive LDNs were enriched for MPO and associated with inflammatory biomarkers, Sequential Organ Failure Assessment (SOFA) scores, vasopressor use, and mechanical ventilation. R406 exposure reduced selected LPS-induced phenotypic responses and LDN MPO release ex vivo [12]. These data connect a cell-specific abundance marker to clinical severity and laboratory pharmacological responses, while neither establishing causality from abundance nor demonstrating treatment efficacy in patients.

SYK also participates in human monocyte signaling. Shalova et al. combined mechanistic experiments in human monocytes with observations of CD16-expressing subsets from patients with sepsis [62]. CD16-dependent signaling through SYK modified the TRIF arm of TLR4 signaling, promoting interferon regulatory factor 3 and signal transducer and activator of transcription 1 responses and expression of interferon beta, C-C motif chemokine ligand 5, and C-X-C motif chemokine ligand 10. CD16 also increased negative regulators of the MyD88-dependent pathway [62]. Corresponding transcriptional changes in patient-derived monocytes support relevance to sepsis-associated immune reprogramming, while remaining distinct from an interventional demonstration of SYK-dependent clinical outcomes.

Macrophage responses provide an additional example of receptor-dependent integration through SYK. The CD300b receptor experiments described in Section 5.1 provide murine evidence for this integration [59]. At the same time, SYK-dependent innate immune activation is closely interconnected with phagocyte oxidative mechanisms, particularly NADPH oxidase 2-mediated ROS generation. This pathway is essential for antimicrobial defense and microbial killing, but excessive or prolonged ROS production may also contribute to collateral tissue injury during systemic inflammation [63]. Representative experimental, translational, and clinical studies investigating SYK expression, activation, and pharmacological modulation in sepsis are summarized in Supplementary Table S2.

Across these populations, oxidative activity, degranulation, NET formation, adhesion, and cytokine reprogramming provide different links to organ injury. The compartmental distinction parallels tumor-cell versus myeloid-cell signaling in cancer, but the outputs in infection must also be evaluated against the requirement for pathogen control [20,32,33,53,61,62].

### 5.3. Endothelial Dysfunction, Microcirculatory Alterations, and Organ Injury

Endothelial dysfunction is a central component of sepsis-associated organ injury, linking systemic inflammation to increased vascular permeability, leukocyte adhesion, coagulation activation, heterogeneous tissue perfusion, and impaired oxygen delivery. Although multiple inflammatory mediators contribute to endothelial damage, accumulating experimental evidence indicates that SYK can directly influence endothelial barrier integrity in addition to its effects in circulating immune cells. This dual involvement provides a mechanistic connection between immune-cell activation and vascular dysfunction during sepsis.

Shadab et al. demonstrated a direct endothelial mechanism using human pulmonary artery endothelial cells and murine sepsis models [9]. SYK associated with VE-cadherin, and SYK depletion or a kinase-defective construct reduced thrombin-induced phosphorylation of VE-cadherin at Tyr658 and Tyr685 and barrier disruption. The Tyr658/Tyr685 sites belong to VE-cadherin rather than SYK. Endothelial-specific SYK deletion protected mice against loss of VE-cadherin and pulmonary vascular leakage after cecal ligation and puncture. Fostamatinib also attenuated lung injury and improved murine survival [9]. Convergent genetic, biochemical, and pharmacological evidence strengthens the causal interpretation of endothelial SYK function, while the survival result remains preclinical.

Loss of endothelial barrier function contributes to intravascular fluid escape, interstitial edema, altered rheology, and reduced effective capillary perfusion. In NOSTRADAMUS, sublingual microvascular imaging in 34 patients with sepsis and 17 controls identified reduced small-capillary density and associations between microvascular parameters and disease severity [64]. In the prospective observational EDGE study, sublingual videomicroscopy in 299 emergency-department patients with suspected infection or sepsis identified associations between capillary density, glycocalyx-related measurements and subsequent disease progression [65]. These observations extend microvascular assessment to early clinical presentation without establishing a SYK-specific biomarker or a causal treatment effect. Both investigations provide clinical vascular context rather than evidence of SYK-specific causality.

Endothelial injury in septic shock is also closely linked to disturbed hemostasis. In the ProCESS cohort, Hou et al. prospectively evaluated circulating markers of endothelial permeability and coagulation in patients with septic shock and found that biomarkers reflecting endothelial activation, permeability, and hemostatic dysfunction were significantly higher in nonsurvivors [66]. The coexistence of increased permeability, endothelial activation, platelet–leukocyte interactions, and coagulation abnormalities favors microvascular thrombosis and heterogeneous capillary obstruction. SYK may contribute to several components of this process through Fc receptor- and integrin-dependent leukocyte activation, platelet signaling, oxidative responses, and endothelial barrier disruption, although the specific contribution of endothelial or platelet SYK to human sepsis-associated immunothrombosis remains insufficiently defined.

At the organ level, murine pharmacological evidence links SYK-related signaling with dysfunction extending beyond the pulmonary vasculature. In sepsis-associated AKI, SYK inhibition reduced neutrophil oxidative responses and improved renal function in mice [8]. More recently, Verra et al. demonstrated increased SYK phosphorylation in cardiac tissue following polymicrobial sepsis and showed that administration of PRT062607 one hour after sepsis induction improved both systolic and diastolic cardiac function [10]. PRT062607 also preserved pulmonary artery and aortic blood flow and reduced serum indices of renal dysfunction, hepatocellular injury, skeletal muscle injury, and generalized cellular damage [10]. These effects were accompanied by reduced cardiac IKK phosphorylation, lower NLRP3 abundance, and suppression of systemic inflammatory mediators, supporting investigation of SYK-related inflammatory pathways across organ compartments without establishing human therapeutic efficacy [10].

These studies connect cellular SYK mechanisms with organ injury experimentally. The human microcirculatory and endothelial-biomarker studies establish the clinical importance of vascular dysfunction, but do not directly measure or prove a causal role for SYK [64,65,66].

### 5.4. SYK Across the Immunological Phases of Sepsis

Sepsis-associated immune dysfunction is not adequately described by a strictly sequential transition from an early hyperinflammatory state to a late immunosuppressive phase. Proinflammatory and immunosuppressive programs can coexist from the early stages of disease, with their relative dominance varying according to pathogen burden, host characteristics, tissue compartment, treatment, and time from infection. This temporal and cellular heterogeneity is particularly relevant to SYK because the same kinase participates in antimicrobial effector responses, inflammatory amplification, and signaling mechanisms that can contribute to immune reprogramming.

During early infection, receptor-dependent SYK activation can support host defense by promoting phagocytosis, oxidative burst, degranulation, cytokine production, and recruitment of innate immune cells [2,20,61]. Excessive or sustained activation, however, can amplify ROS generation, endothelial injury, inflammasome signaling, and organ dysfunction [8,9,10]. The biological value of SYK activation therefore depends on whether its activity contributes predominantly to pathogen clearance or to disproportionate inflammatory and tissue-damaging responses. This distinction also complicates therapeutic inhibition, because suppression of SYK during a strongly inflammatory phenotype may attenuate injury, whereas equivalent inhibition in patients with impaired antimicrobial immunity could potentially be detrimental.

Endotoxin tolerance provides a mechanistic framework for understanding the immunoreprogrammed state that develops during sepsis. Repeated exposure of monocytes and macrophages to LPS reduces subsequent proinflammatory cytokine responses despite continued receptor stimulation. Importantly, SYK may participate in this suppressive phenotype rather than simply becoming inactive. In endotoxin-tolerant macrophage experiments, Wu et al. demonstrated that sialic acid-binding immunoglobulin-like lectin 1 (Siglec-1) was increased in endotoxin-tolerant macrophages and associated with an adaptor-dependent SYK pathway that promoted transforming growth factor beta 1 production; pharmacological SYK inhibition significantly attenuated this response [67]. These findings suggest that SYK signaling can be redirected toward immunoregulatory outputs under tolerant conditions.

Evidence from patients indicates that immune reprogramming is not restricted to the late phase of sepsis. Baghela et al. analyzed whole-blood transcriptomes from 348 patients and 44 controls and identified five endotypes with different severity-associated immune signatures at first clinical presentation [68]. The analysis supports early heterogeneity of inflammatory and immune-suppressed states but does not directly establish SYK activation or validate a SYK biomarker.

Keramati et al. used an in vivo LPS challenge in human volunteers to identify an acute monocyte inflammatory program followed by reduced type I interferon-responsive expression, altered myelopoiesis, and attenuated responses to a second challenge one week later; related transcriptional changes were found in bacterial sepsis cohorts [69]. These observations concern immune-state dynamics, not a direct test of SYK function. Together with the neutrophil-expression findings [12], they provide a rationale for evaluating SYK measurements longitudinally, rather than assuming that abundance identifies a single inflammatory phase.

Longitudinal SYK assessment in defined populations is therefore a testable biomarker-development strategy, not an established clinical application. In contrast to malignant-cell signaling dependence, an infection-associated readout must be interpreted alongside pathogen burden, immune competence, and organ dysfunction before it can inform a decision to inhibit the pathway.

## 6. Shared and Divergent Mechanisms in Malignancies and Sepsis

The comparison of malignancies and sepsis is most informative when the receptor–cell–time relationship is made explicit. In B-cell malignancies, tonic or chronic active BCR signaling can support persistence of a malignant clone, while ITK–SYK fusion provides a receptor-independent oncogenic scaffold [25,26,27,39,40]. In sepsis, Fc receptors, CLRs, integrins, and adaptor-mediated cooperation with TLR4 connect changing microbial stimuli to heterogeneous immune and vascular responses [2,55,58,59]. Solid tumors bridge these settings analytically because SYK in malignant epithelial cells can have a different function from SYK in infiltrating myeloid cells [32,33,45,53]. Supplementary Table S3 summarizes the resulting shared and divergent features without treating the two disease contexts as biologically equivalent.

PI3K–AKT, MAPK, and NF-κB are shared downstream modules, but their engagement does not imply a common disease mechanism. In B-cell malignancies, persistent BCR–SYK input can sustain survival, metabolic activity, and clonal expansion [25,26,27]. In innate cells, receptor combinations configure cytokine production, antimicrobial functions, and inflammatory amplification [22,55,59]. The scaffold also differs: BLNK–BTK–PLCγ2 is prominent in B cells, whereas CARD9–BCL10–MALT1 couples selected CLR signals to innate inflammatory outputs [21,22]. A similar downstream readout must therefore be interpreted through the upstream receptor and cellular compartment.

Cell survival and cytoskeletal regulation illustrate the divergent outputs. SYK-directed treatment can modify survival-associated signaling in CLL [34], whereas experimental inhibition can impair malignant-cell survival or AML stem-cell metabolic function [25,35]. In solid tumors, SYK isoforms may either promote or suppress proliferation and invasion [45,49,52]. In ovarian cancer, SYK-linked cytoskeletal phosphorylation favors motility and invasion [50]. In neutrophils, related adhesion and cytoskeletal machinery supports phagocytosis, migration, degranulation, and oxidative responses [20,61]. Inhibiting a malignant-cell dependency and attenuating an excessive innate-cell response are consequently different objectives, even when some downstream proteins overlap.

Myeloid reprogramming provides a second point of convergence. Genetic and pharmacological studies of tumor-associated macrophages implicate SYK in immunosuppressive programs that can favor tumor growth and restrict T-cell responses [32,33]. In infection-related models, CD16–SYK–TLR4 cooperation and Siglec-1-associated signaling can reshape monocyte or macrophage responses toward different inflammatory or immunoregulatory outputs [62,67]. These programs should not be equated: tumor immune escape and sepsis-associated immune dysfunction differ in initiating stimuli, host-defense requirements, and supporting evidence. Their comparison instead highlights how a shared kinase can be redirected within myeloid cells.

Temporal behavior further separates the biomarker questions. Tonic BCR signaling is maintained without acute receptor crosslinking, although malignant cells remain responsive to microenvironmental stimulation [25,26,27,29]. In the nonhuman primate sepsis study, neutrophil SYK abundance was highest early after infection and changed across subsequent time points; this was an expression trajectory, not a phosphorylation time course [11]. Human endotoxemia and tolerance studies show that inflammatory and suppressive programs can overlap or persist, but do not independently establish the kinetics of SYK activity [67,68,69]. Serial measurements must therefore distinguish abundance changes, population shifts, and phosphosite-specific signaling.

Some mechanisms remain disease-specific. Oncogenic fusions, isoform-dependent tumor suppression, and homologous recombination-associated treatment resistance have no demonstrated counterpart in sepsis [39,40,45,49,51]. Endothelial VE-cadherin disruption and acute organ dysfunction are central to experimental sepsis, whereas the human microcirculatory observations provide downstream clinical context rather than direct SYK causality [9,10,64,65,66]. Similarly, early therapeutic trials in selected malignancies provide human interventional evidence, while the sepsis literature contains animal protection, human expression associations, and ex vivo pharmacological responses [8,9,10,11,12,31,70,71,72]. This evidentiary asymmetry is part of the comparison, not a gap that shared-pathway language can overcome.

The translational implication is a disease-specific interpretation of the measured endpoint. Tumor-cell p-SYK may support a hypothesis of signaling dependence, whereas total neutrophil SYK may identify a population associated with acute disease severity; neither is automatically a validated predictor of treatment benefit [12,38,43]. Total tissue expression can also mask opposing tumor-cell and immune-cell functions [32,33,49,53]. The laboratory discussion therefore separates analyte identity, cellular source, and sampling context, and the therapeutic discussion separates malignant dependency from modulation of host responses.

## 7. Laboratory Assessment and Biomarker Potential

Translation of SYK measurements requires a defined analyte and a defined cellular source. Transcript abundance, total protein, the percentage of SYK-positive cells, phosphosite-specific signal, and functional pathway responses provide different information [12,25,38]. In oncology the sample may contain malignant and infiltrating cells; in septic blood the changing proportions of leukocyte subsets are themselves biologically informative. Section 7.1 describes analytical approaches, and Section 7.2 distinguishes disease characterization, prognostic association, and prediction of treatment response.

### 7.1. Analytical Approaches for SYK Assessment

SYK can be assessed at the transcript, total protein, phosphorylated protein, and pathway-activity levels using complementary molecular and cellular techniques. At the transcriptional level, reverse transcription quantitative polymerase chain reaction (RT-qPCR) provides a relatively sensitive approach for measuring SYK messenger RNA and can also distinguish specific transcript variants when appropriately designed primers are used. In oral squamous cell carcinoma, Ogane et al. combined quantitative RT-PCR with protein-based methods and demonstrated concordant reductions in SYK messenger RNA and protein expression in malignant cells, illustrating the value of integrating transcriptional and protein measurements rather than relying on a single analytical level [73]. Transcript abundance, however, does not directly indicate catalytic activity because activation of SYK is predominantly regulated through receptor recruitment, conformational changes, and phosphorylation.

Western blotting provides information on total SYK protein and, when phospho-specific antibodies are used, on individual phosphorylation sites associated with kinase regulation [14]. This approach can differentiate changes in protein abundance from changes in phosphorylation. The principal limitations of Western blotting include the need for relatively large quantities of cellular material, limited cellular resolution, and dependence on antibody specificity and normalization procedures.

Immunohistochemistry (IHC) preserves tissue architecture and permits spatial and subcellular assessment. In Boros et al., p-SYK Tyr323 staining in 70 AML marrow biopsies associated with unfavorable outcome; its use as an activity surrogate was supported by comparison with activation-loop Tyr525/Tyr526 phosphorylation in cell lines [38]. The inhibitor-sensitivity experiments were performed in those cell lines, not validated as a patient treatment-response test. Fixation, antigen preservation, antibody specificity, scoring, and intratumoral heterogeneity influence results. Total SYK IHC and phosphosite-specific IHC therefore require separate analytical and clinical validation. Zhang et al. assessed total SYK across T- and NK-cell lymphoid disorders using multiparameter flow cytometry and tissue immunohistochemistry [74]. Their series showed marked subtype dependence, with higher expression in several precursor and innate-lineage neoplasms and generally low expression in many mature T-cell lymphomas. These measurements concern protein abundance and cellular attribution; they do not establish SYK phosphorylation or therapeutic dependence.

Flow cytometry attributes intracellular SYK abundance to phenotypically defined populations. Conventional intracellular staining in the human sepsis study measured total SYK and SYK-positive neutrophil subsets; it did not measure phosphorylated SYK [12]. Phospho-flow adds site-specific phosphorylation measurements to immunophenotyping [75]. Total kinase abundance, phosphorylation and catalytic output remain distinct endpoints, even when measured within a shared experimental workflow. NanoBRET assays developed for SYK gain-of-function variants provide a complementary measure of cellular inhibitor target engagement, which must be distinguished from kinase activity and clinical biomarker performance [76].

Proteomic and phosphoproteomic approaches provide a broader assessment of SYK-associated signaling by identifying phosphorylation events and interacting proteins rather than measuring a single kinase endpoint. Kassouf et al. combined mass-spectrometric analysis of E-cadherin/catenin phosphorylation with biochemical and epithelial-model experiments linking SYK to junctional stability and reduced breast-cancer invasion [77]. Such analyses require perturbation and biochemical validation to distinguish candidate regulated sites from direct kinase substrates. Their cost, technical complexity, specialized bioinformatics requirements, and limited standardization restrict routine clinical use.

Supplementary Table S4 summarizes these analytical approaches. Whole-blood analysis, density-separated neutrophils, purified leukocytes, and bulk tissue should not be treated as equivalent sample matrices: isolation and changes in population abundance can affect the signal attributed to SYK [11,12,33,53]. For phospho-assays, the sampling interval relative to infection or stimulation, processing delay, fixation/permeabilization, phospho-epitope preservation, antibody clone and site specificity, and normalization or gating must be documented [75]. Protein-lysate assays require appropriate loading and total-protein controls; cellular assays require consistent population definitions and fluorescence controls. A higher site-specific phospho-signal supports a regulatory phosphorylation event, but its functional meaning depends on the site and supporting pathway evidence [14,38].

### 7.2. Diagnostic, Prognostic, and Predictive Biomarker Potential

A prognostic association describes outcome or severity, whereas a predictive biomarker must identify differential benefit from an intervention. The SYK literature contains molecular characterization, outcome associations, ex vivo drug-sensitivity observations, and early therapeutic trials; these categories should not be conflated.

As a diagnostic biomarker in malignancies, SYK is most informative when interpreted within a defined molecular or histopathological context rather than as a standalone marker. Subtype-dependent total SYK abundance has been documented in T- and NK-cell lymphoid disorders [74]. In DLBCL models, experimental HSP110 perturbation modified SYK phosphorylation, providing mechanistic evidence without establishing clinical diagnostic utility [25]. Separately, the ITK–SYK fusion represents a specific molecular alteration in a subset of PTCL [39]. However, SYK expression is not restricted to malignant cells and can also be detected in normal hematopoietic and non-hematopoietic tissues [1,16]. Its heterogeneous and sometimes opposing expression patterns in solid tumors further limit its usefulness for general cancer detection. Accordingly, SYK-related measurements may contribute to molecular characterization of selected tumors, but current evidence does not support their use as broadly applicable standalone diagnostic tests.

The prognostic potential of SYK is supported by selected human cohorts. In AML, p-SYK Tyr323 IHC associated with unfavorable outcome independently of age, cytogenetic risk, and leukocyte count [38]. Greater BCR-induced phosphorylation responsiveness involving SYK associated with poorer survival in MCL [43], while distinct hepatocellular carcinoma isoform patterns associated with recurrence and survival [49]. Conversely, compartment-specific SYK abundance in breast and lung carcinoma associated with lymphocytic infiltration and more favorable outcomes [53]. These are context-specific observational findings, not a universal prognostic rule or an established clinical assay.

Evidence for treatment-response assessment includes mechanistic and ex vivo studies. In AML cell lines, higher p-SYK at Tyr323 and Tyr525/Tyr526 correlated with sensitivity to small-molecule inhibitors, while the separate marrow cohort supported a prognostic association [38]. In BCR-dependent DLBCL models, Chen et al. found that pharmacological SYK inhibition and genetic SYK depletion increased CXCR4 expression; PI3K inhibition produced related effects, and SYK blockade enhanced CXCL12-directed migration [78]. The study included cell lines and six primary tumor preparations. CXCR4 induction is a candidate downstream response to pathway perturbation, not a direct SYK activity measurement or a clinically validated predictor of treatment benefit. The mivavotinib trial provided clinical response data without establishing an expression- or phosphorylation-based predictive test [70]. Analysis of 48 primary AML samples exposed in vitro to five inhibitors also showed heterogeneous sensitivity and associated transcriptional profiles [79]. Such observations support candidate response signatures; they do not establish a prospectively validated predictor of clinical treatment benefit.

In sepsis, the human neutrophil study and preceding nonhuman primate report associate SYK abundance with severity or organ-dysfunction measures [11,12]. These findings do not establish diagnostic discrimination from sterile inflammation, trauma, infections without sepsis, or noninfectious critical illness. Validated decision thresholds, reference intervals, independent validation cohorts, and prospective performance measures are currently lacking. The human data therefore support severity-related biomarker investigation rather than a validated diagnostic application.

Predictive applications in sepsis remain hypotheses. R406 attenuated selected activation-associated phenotypes and LDN MPO release in patient-derived neutrophils ex vivo [12], while cellular and murine studies showed pharmacological effects on inflammatory and organ-injury endpoints [8,9,10,61]. Neither response in isolated cells nor protection in an animal model establishes that a patient’s SYK abundance or phosphorylation predicts benefit from systemic treatment. Clinical evaluation would need an independently validated assay, disease-stage definition, and an intervention study relating the biomarker to treatment benefit.

## 8. Therapeutic Implications of SYK Modulation

The central position of SYK within receptor-dependent signaling has generated considerable interest in its pharmacological modulation. In malignancies, inhibition of SYK has been explored primarily as a strategy to disrupt survival and proliferative signaling in SYK-dependent tumor cells, whereas in sepsis the therapeutic rationale is based on limiting excessive inflammatory, oxidative, and organ-damaging responses. These different objectives require distinct approaches to patient selection, treatment timing, and interpretation of pharmacodynamic effects.

### 8.1. SYK Inhibition in Malignancies

Mivavotinib, a dual SYK/FLT3 inhibitor, was evaluated in a nonrandomized phase I study with updated results in 124 patients with relapsed/refractory lymphoma, including 89 with DLBCL [70]. Responses occurred in 43/124 and 26/89 patients, respectively; the response-evaluable estimates were 43/95 (45%) and 26/69 (38%). Grade ≥ 3 treatment-emergent adverse events were reported in 119/124 patients (96%), whereas grade ≥ 3 treatment-related adverse events occurred in 94/124 patients (76%). These findings demonstrate clinical activity with substantial toxicity and do not isolate the contribution of SYK inhibition or establish comparative efficacy.

Entospletinib has also been studied with the anti-CD20 antibody obinutuzumab in a single-arm phase I/II investigation [71]. Of 24 enrolled patients with relapsed/refractory B-cell malignancies, 23 were evaluable after one eligibility exclusion, and efficacy was assessed in 21 patients with CLL. Fourteen responded (66.7%), including three complete responses (14.3%); median event-free survival was 24 months. These combination-treatment findings cannot be attributed to entospletinib alone and do not establish superiority to another regimen. The pharmacodynamic observations reported by Lam et al. [34] arose from a related correlative analysis of this trial and do not constitute an independent clinical cohort.

In a single-arm phase Ib/II study of 53 patients with previously untreated AML, Walker et al. combined entospletinib with induction chemotherapy and reported a composite complete response rate of 70% [72]. Higher baseline HOXA9 and MEIS1 expression associated with improved survival. Because patients received chemotherapy and there was no randomized comparator, the contribution of entospletinib and the predictive value of the expression signature remain unresolved [72]. The AML cell-line p-SYK findings and patient-derived ex vivo sensitivity profiles provide supporting hypotheses, rather than confirmation of that clinical predictive relationship [38,79].

Cerdulatinib combines SYK and Janus kinase (JAK) inhibition. In a single-arm phase IIa PTCL study, 65 patients were treated and 58 were efficacy-evaluable; the overall response rate was 36.2%, including 51.9% in the angioimmunoblastic/T-follicular-helper subgroup [31]. Clinical activity with a dual inhibitor supports investigation of cooperating pathways but cannot be attributed exclusively to SYK. This intentional multi-target activity should also be distinguished from unintended off-target effects in mechanistic studies. Preclinical MCL studies with luxeptinib further support investigation of combined BTK/SYK pathway inhibition, including effects on BCR signaling and apoptotic susceptibility [80].

Across these trials, clinical activity does not make SYK inhibition a universal cancer strategy or validate total SYK as a selection test [31,70,71,72]. Molecular dependence, tumor-cell versus immune-cell effects, combination partners, and treatment toxicity remain relevant to disease-specific development.

### 8.2. Experimental SYK Modulation in Sepsis

The sepsis objective is to reduce injurious host responses while preserving pathogen control, rather than to eliminate a malignant signaling dependency. Pharmacological murine experiments, endothelial genetic studies, and human neutrophil ex vivo work support investigation of this balance [8,9,10,12,61]. These findings provide mechanistic support of differing strength, but do not establish clinical efficacy.

R406 reduced renal dysfunction, neutrophil oxidative abnormalities, and dendritic-cell cytokine production in the Al-Harbi murine study [8]. In an Fcγ receptor IIb-deficient lupus mouse model, Issara-Amphorn et al. administered a SYK inhibitor before polymicrobial sepsis and reported reduced inflammatory cytokines, organ injury, and mortality [81]. The pretreatment schedule and lupus-prone immunological background limit extrapolation to treatment of unselected patients after sepsis onset. Both studies support experimental pharmacological modulation; their effects cannot be assigned exclusively to SYK without considering inhibitor selectivity and model-specific biology.

The vascular compartment represents another therapeutically relevant target. Shadab et al. combined pharmacological inhibition with endothelial-specific genetic depletion of SYK and demonstrated that reduction of endothelial SYK function preserved VE-cadherin, decreased vascular permeability, neutrophil infiltration, and pulmonary edema, and attenuated sepsis-associated lung injury [9]. Administration of fostamatinib after induction of polymicrobial sepsis also reduced lung injury and improved survival [9]. The concordance between genetic and pharmacological approaches strengthens the evidence that the observed vascular protection was related, at least in part, to SYK-dependent mechanisms rather than exclusively to nonspecific drug effects.

Verra et al. administered PRT062607 one hour after cecal ligation and puncture in mice [10]. Reduced cardiac SYK phosphorylation accompanied improved systolic and diastolic function, preserved systemic and pulmonary flow, and reduced biochemical indices of renal, hepatic, and muscular injury. Reduced IKK phosphorylation and NLRP3 abundance were measured; NLRP3 protein reduction should not itself be equated with demonstration of reduced inflammasome assembly or activity [10]. Post-insult treatment and a phosphorylation readout strengthen translational relevance and target-engagement interpretation, but a pharmacological design without independent genetic confirmation does not exclude all off-target contributions.

Human ex vivo work provides a complementary but bounded test. R406 attenuated selected injurious effector responses in LPS-stimulated healthy-donor neutrophils while preserving several measured functions [61]. Patient-derived neutrophils also showed altered activation-associated phenotypes and reduced LDN MPO release [12]. These findings identify measurable pharmacodynamic responses under laboratory conditions; they do not establish systemic therapeutic benefit, adequate microbial clearance, or the safety of inhibiting SYK in patients with sepsis.

Therapeutic translation is complicated by the physiological requirement for SYK in Fc receptor, integrin, CLR, phagocytic, oxidative, and other antimicrobial signaling pathways [2,20]. The same kinase activity that contributes to endothelial injury and excessive inflammation may therefore remain necessary for effective pathogen clearance. Furthermore, the immunological state of sepsis changes over time, and patients with hyperinflammatory phenotypes may respond differently from those with established immune dysfunction. The experimental evidence consequently supports evaluation of cell-specific SYK activation, treatment timing, degree of kinase inhibition, and pharmacodynamic biomarkers as central variables in future translational studies rather than indiscriminate systemic suppression of SYK activity.

## 9. Limitations and Perspectives

Interpretation is limited by unequal evidence maturity and substantial heterogeneity within each context. Oncology includes human cohorts and early interventional studies, but findings differ between hematological neoplasms, solid tumors, and malignant versus immune compartments [31,38,49,53,70,71,72]. Sepsis has causal evidence predominantly from cellular and murine models, together with a small nonhuman primate series and limited human observational/ex vivo work [8,9,10,11,12,61,62]. Neither model protection nor severity associations establish a clinically ready diagnostic, prognostic, predictive, or therapeutic application in sepsis.

Analytical heterogeneity is a second limitation. Bulk transcript or total-protein measurements can reflect cellular composition and do not establish activation. Phosphorylation assays depend on site selection, collection time, processing, fixation/permeabilization, antibody specificity, phospho-epitope preservation, and normalization or gating [14,38,75]. These differences restrict direct comparison between studies and may obscure opposing cellular signals. The distinction between conventional total-SYK flow cytometry and phospho-flow is particularly important when interpreting the neutrophil studies [11,12].

The narrative design permits selection bias and limits search reproducibility, as described in Section 2. Heterogeneous models, stimulation protocols, disease definitions, specimen types, treatment settings, and outcome measures further restrict comparison. The representative supplementary evidence tables are not an exhaustive inventory or formal quality assessment. Evidence published after August 2026 is outside the documented search window.

Future investigations should prioritize longitudinal, cell-specific, and activation-state-dependent assessment of SYK. In sepsis, serial evaluation of SYK expression and phosphorylation across defined immune-cell populations may help determine whether distinct patterns correspond to hyperinflammatory, immunoregulatory, or immunosuppressive phenotypes. Integration with inflammatory biomarkers, organ-dysfunction indices, immune-function assays, and clinical severity scores could clarify whether SYK provides independent diagnostic or prognostic information beyond currently available parameters, including composite biomarker-based approaches to sepsis risk stratification [82].

In malignancies, research should focus on identifying molecular contexts in which tumor cells are functionally dependent on SYK signaling. Isoform-specific expression, phosphorylation status, upstream receptor activity, interacting signaling pathways, and tumor-microenvironment composition may provide greater biological and therapeutic information than total SYK abundance alone. Multiparametric profiling could support more precise selection of patients for SYK-directed therapy and help distinguish tumors in which kinase inhibition is potentially beneficial from those in which SYK retains tumor-suppressive or immune-supportive functions.

Pharmacological interpretation also requires caution. R406/fostamatinib can affect kinases beyond SYK, and biochemical target engagement alone does not prove that every downstream outcome is target-specific [10,25,61]. Genetic depletion, kinase-defective constructs, and convergent approaches strengthen attribution, as illustrated by endothelial and macrophage studies [9,32,33]. Dual inhibitors such as cerdulatinib intentionally act on more than one pathway [31]. In sepsis, future intervention studies should establish dose, timing, duration, effects on microbial clearance and immune competence, and biomarker-defined treatment interactions before clinical benefit is inferred.

## 10. Conclusions

Comparing malignancies and sepsis shows that the biological meaning of SYK arises from the receptor and cellular compartment in which it operates, the measured activation state, and the duration of the response. Sustained malignant-cell signaling, isoform-dependent tumor restraint, tumor-associated myeloid programs, and stimulus-driven host defense are distinct uses of shared signaling machinery. Their differences explain why total expression, phosphorylation, and functional response cannot be assigned a common biomarker meaning across the two disease settings.

Oncology provides the more mature mechanistic, translational, and selected clinical therapeutic evidence, although biomarker-guided benefit remains incompletely established. In sepsis, human severity associations and ex vivo responses complement experimental causality but do not yet establish a validated biomarker or treatment. Translation should link a cell- and site-defined measurement to a disease-specific decision: whether a malignant population depends on SYK, or whether modulating an infection-associated response can reduce injury while preserving host defense.

## Data Availability

No new data were created or analyzed in this study. Data sharing is not applicable to this article.

## Supplementary Materials

The following supporting information can be downloaded at: https://www.mdpi.com/article/10.3390/biom16101529/s1, Table S1: Representative Experimental, Translational, and Clinical Evidence for SYK in Malignancies; Table S2: Experimental and Clinical Evidence Supporting the Involvement of SYK in Sepsis; Table S3: Comparative Framework of SYK Biology in Malignancies and Sepsis; Table S4: Analytical Approaches for SYK Assessment and Their Potential Biomarker Applications. References cited in Tables S1–S4 correspond to the reference list of the main manuscript.
