# Supplementary material for: Spleen Tyrosine Kinase in Malignancies and Sepsis: Pathophysiological Roles and Biomarker Potential—A Narrative Review
Source: Biomolecules. 2026 Oct 20;16(10):1529. doi: 10.3390/biom16101529 (PMC13702482; doi:10.3390/biom16101529)
Supplement: Supplementary file 1 [file biomolecules-16-01529-s001.zip › Supplementary_Table_S1.pdf]

Table S1. Representative Experimental, Translational, and Clinical Evidence for SYK in Malignancies

Malignancy / tumor type	Study design/model	Evidence level / type	Sample size, where applicable	SYK parameter	Main finding	Biological / clinical relevance	Ref.
**DLBCL**	BCR-dependent cell lines and primary tumor preparations; genetic and pharmacological SYK/PI3K perturbation	Mechanistic in vitro + human ex vivo	Six primary tumor preparations; model-dependent cell-line experiments	CXCR4 transcript/surface abundance and CXCL12-directed migration after pathway blockade	SYK inhibition/depletion increased CXCR4; SYK blockade enhanced CXCL12-directed migration.	Candidate downstream pharmacodynamic/compensatory response; not direct SYK activity or a validated clinical predictive test.	[78]
**Relapsed / refractory B-cell lymphoma, including DLBCL**	Updated nonrandomized phase I mivavotinib (TAK-659/CB-659)	Interventional clinical trial	124 lymphoma patients, including 89 DLBCL; response-evaluable: 95 overall, 69 DLBCL	Intentional dual SYK/FLT3 inhibition	Responses: 43/124 overall; 26/89 DLBCL. Evaluable ORR: 45% (43/95) and 38% (26/69). Grade ≥3 adverse events: 96% treatment-emergent and 76% treatment-related.	Clinical activity with substantial toxicity; no randomized efficacy comparison or isolated SYK attribution.	[70]
**CLL / relapsed-refractory B-cell malignancies**	Single-arm phase I/II entospletinib–obinutuzumab combination	Interventional clinical trial	24 enrolled; 23 evaluable after an eligibility exclusion; 21 CLL efficacy-evaluable	SYK-directed combination therapy; clinical response	CLL: 14/21 responses (66.7%), including 3 complete responses (14.3%); median event-free survival 24 months.	Combination effect cannot be attributed to entospletinib alone; related to the Lam correlative report, not an independent cohort.	[71]
**MCL**	Primary-cell phospho-flow with BCR stimulation and follow-up	Human cellular + clinical observational	30 patients (19 at diagnosis, 11 at relapse); 10 healthy donors	Basal and induced phosphorylation responses	Higher SYK response associated with shorter OS; signaling was heterogeneous.	Prognostic hypothesis; stimulus response differs from total abundance.	[43]
**AML**	Cell lines, primary blasts ex vivo, pathway rescue, metabolic assays and NSG/J engraftment experiments	Mechanistic cellular + human ex vivo + preclinical murine	Primary-blast phospho-flow: 7 patients; mitochondrial transcript/mass assays: 3; engraftment: one primary specimen, 5 mice/group; overall unique donor total NR	R406-associated pathway perturbation and leukemia stem-cell oxidative metabolism	Reduced oxidative metabolism, clonogenic potential and LSC-enriched populations; ex vivo blast treatment reduced subsequent murine engraftment.	Pharmacological/metabolic target rationale with downstream rescue experiments; not patient treatment efficacy.	[35]

Malignancy / tumor type	Study design/model	Evidence level / type	Sample size, where applicable	SYK parameter	Main finding	Biological / clinical relevance	Ref.
**AML**	Cell-line inhibitor assays; marrow-biopsy IHC and survival analysis	Mechanistic in vitro + clinical observational	70 marrow biopsies; 17 AML cell lines	p-SYK Tyr323 IHC; Tyr323 and Tyr525/Tyr526 in cell lines	Higher patient p-SYK associated with worse outcome; cell-line p-SYK associated with drug sensitivity.	Patient prognosis and experimental sensitivity are distinct findings.	[38]
**AML**	Primary AML cells exposed in vitro to five kinase inhibitors; transcriptomics	Translational human ex vivo	48 primary AML samples	Drug sensitivity and gene-expression signatures	Marked interpatient sensitivity variation and distinct transcriptional profiles.	Candidate ex vivo response signatures; inhibitor profiles differ; no clinical predictive validation.	[79]
**AML**	Entospletinib plus induction chemotherapy, single-arm phase Ib/II	Interventional clinical trial	53 patients: 12 phase Ib + 41 phase II	SYK-targeting combination; HOXA9/MEIS1 expression	Composite complete response 70%; higher HOXA9/MEIS1 associated with survival.	Chemotherapy contribution and predictive treatment interaction cannot be isolated.	[72]
**PTCL**	Fusion detection in lymphoma specimens	Human molecular/tissue study	PTCL unspecified: 30; AITL: 9; ALK-negative ALCL: 7	ITK–SYK fusion	Fusion detected in 5/30 unspecified PTCL cases.	Defined oncogenic alteration in a subset; not universal PTCL biology.	[39]
**PTCL**	Conditional ITK–SYK expression and mechanistic models	Mechanistic + preclinical murine	N/A	Constitutive fusion-kinase signaling	ITK–SYK mimicked proximal TCR signaling and drove lymphoma in mice.	Causal oncogenic model distinct from stimulus-dependent host signaling.	[40]
**T- and NK-cell lymphoid disorders**	Multiparameter flow cytometry and IHC	Human cellular/tissue observational	91 patients assessed by flow; 53 by IHC; 16 assessed by both methods (overlapping denominators)	Total SYK protein abundance and cellular attribution	Marked subtype dependence: higher abundance in several precursor/innate-lineage neoplasms; generally low expression in many mature T-cell lymphomas.	Abundance benchmarking; no direct phosphorylation or therapeutic-dependence measurement.	[74]
**PTCL**	Single-arm phase IIa cerdulatinib	Interventional clinical trial	65 treated; 58 efficacy-evaluable; treated AITL/TFH 29, PTCL-NOS 11, other 25	Intentional dual SYK/JAK inhibition	ORR 36.2% among efficacy-evaluable patients; AITL/TFH ORR 51.9%.	Human clinical activity cannot be assigned to SYK alone.	[31]
**Breast cancer**	Primary tissues and cell lines; methylation and demethylation	Human tissue + mechanistic in vitro	37 primary tumors; 20 cell lines	SYK promoter methylation and transcript expression	Hypermethylation in 12/37 tumors and 6/20 cell lines; demethylation restored expression.	Epigenetic silencing of a candidate suppressor; abundance, not an activation assay.	[46]

Malignancy / tumor type	Study design/model	Evidence level / type	Sample size, where applicable	SYK parameter	Main finding	Biological / clinical relevance	Ref.
**Breast cancer / TNBC**	Chemical genetic screen, multi-omics, cellular and xenograft work	Mechanistic in vitro + preclinical	N/A	SYK-linked cell-state dependency and STAT3 signaling	Convergent analyses implicated SYK in a subset of basal-like/TNBC cells.	Tumor-promoting model; multi-target screening compounds require mechanistic triangulation.	[30]
**NSCLC / breast cancer**	Spatial tissue cohorts; cellular TNF signaling; exploratory paired trial biopsies	Observational + mechanistic + exploratory on-treatment effects	NSCLC: 329 and 297; LUAD molecular cohort 182; Breast cancer cohorts: 235 and 139; Paired TNBC biopsies: 3 patients	Compartment-specific total SYK; immune signaling; dual SYK/FLT3 inhibitor	SYK associated with T-cell infiltration/outcome; fewer TILs after TAK-659 following nivolumab in 3 patients.	Immune-supportive context; small sequential-treatment substudy cannot isolate a SYK effect.	[53]
**PDAC**	Archived tissue IHC; SYK restoration in cells; mouse tumor models	Human tissue + mechanistic + preclinical	92 tissue samples; normal tissues excluded from survival analysis	Expression, localization, restoration and promoter methylation	Loss of expression accompanied poor differentiation; restoration reduced growth and invasion.	Tumor-suppressive epithelial context; distinct from macrophage SYK.	[47]
**PDAC**	Myeloid Syk deletion in mice; human expression datasets and slice cultures	Preclinical murine + translational human tissue/ex vivo	Human datasets: 36 paired tumor/normal; scRNA-seq 24 tumors + 11 normal pancreases; slice figures identify 4 patients.	Macrophage expression/p-SYK Tyr348, genetic function and pharmacological modulation	Deletion/inhibition reprogrammed TAMs; R788 countered gemcitabine-associated suppression in models.	Compartment-dependent target rationale; no clinical treatment-efficacy trial.	[33]
**Melanoma**	Melanocyte/melanoma cultures; SYK restoration and p53 perturbation	Mechanistic in vitro	N/A	SYK expression and re-expression	Restoration reduced clonogenicity/invasion and induced senescence-like p53/p21 responses.	Tumor-suppressive cellular function; patient biomarker validation not established.	[48]
**HCC**	Resection cohort, isoform-specific assays, cellular/animal models	Human cohort + mechanistic models	152 patients with paired tumor/nontumor tissue	SYK(L)/SYK(S) isoforms	Full-length and short isoforms had opposing functional and outcome associations.	Total SYK can obscure isoform-specific prognosis and therapeutic meaning.	[49]
**Ovarian cancer**	Recombinant kinase assays; genetic and inhibitor perturbation in cellular and 3D models	Mechanistic in vitro	N/A	p-SYK Tyr525/Tyr526; cortactin/cofilin and actin dynamics	SYK perturbation reduced motility, invasion and actin polymerization.	Tumor-promoting invasion mechanism; no clinical response validation.	[50]

Malignancy / tumor type	Study design/model	Evidence level / type	Sample size, where applicable	SYK parameter	Main finding	Biological / clinical relevance	Ref.
**Ovarian cancer / TNBC**	DNA repair/drug-resistance models; public human survival analyses and patient-derived xenografts	Mechanistic/preclinical + human observational reanalysis	Public survival-analysis cohort sizes and unique PDX-donor total NR; N/A for cellular model component	SYK-dependent homologous recombination	SYK promoted double-strand-break resection/repair and resistance to DNA-targeted treatments.	Disease-specific resistance mechanism; clinical efficacy of targeting it is unproven.	[51]
**Colorectal cancer**	Isoform-specific knockdown in CRC cells	Mechanistic in vitro	N/A	Total versus SYK(L)-selective suppression	Isoform-selective depletion altered viability/apoptosis differently from global suppression.	Molecular-form heterogeneity limits interpretation of total abundance.	[52]
**Diffuse glioma**	Public bulk and single-cell transcriptomic reanalysis; tissue qPCR/IHC	Clinical observational + bioinformatics + human tissue	CGGA 693; TCGA 677 nonduplicate samples; GSE16011 276; Rembrandt 471; local 175 gliomas; separate 37 tumor/peritumoral pairs and 22 normal tissues (overlap not resolved); single-cell donor n NR.	Bulk/cell-specific transcripts and total protein abundance	Higher expression associated with adverse phenotype, macrophage markers and immune checkpoints.	Association and cellular-composition effects; does not prove SYK activation or causality.	[54]
**Oral squamous cell carcinoma**	Primary tissue IHC; cellular transcription, methylation and restoration	Human tissue + mechanistic in vitro	53 tumors; 7 OSCC-derived cell lines	Total transcript/protein expression	Downregulation in 33/53 tumors; re-expression reduced cellular motility/invasion.	Candidate suppressor and tissue association; expression is not kinase activation.	[73]

Note: These representative studies were selected from the malignancy literature discussed in the review; this is not an exhaustive systematic dataset. Evidence levels, cellular compartments, and measured SYK endpoints are distinguished because clinical maturity varies substantially by tumor type. Patient/cohort sizes are given where verified; separate cohorts and assay subsets must not be added together unless explicitly identified as disjoint. N/A, patient/participant sample size not applicable to the mechanistic or animal component; NR, not reported or not reliably retrievable. Phosphosite labels are retained only where verified. Reference numbers correspond to the revised main manuscript. Abbreviations: 3D, three-dimensional; AITL, angioimmunoblastic T-cell lymphoma; AKT, protein kinase B; ALCL, anaplastic large cell lymphoma; ALK, anaplastic lymphoma kinase; AML, acute myeloid leukemia; BCR, B-cell receptor; CGGA, Chinese Glioma Genome Atlas; CLL, chronic lymphocytic leukemia; CMML, chronic myelomonocytic leukemia; CRC, colorectal cancer; DLBCL, diffuse large B-cell lymphoma; FL, follicular lymphoma; FLT3, FMS-like tyrosine kinase 3; HCC, hepatocellular carcinoma; IHC, immunohistochemistry; ITK, interleukin-2-inducible T-cell kinase; JAK, Janus kinase; LUAD, lung adenocarcinoma; MCL, mantle cell lymphoma; NHL, non-Hodgkin lymphoma; NOS, not otherwise specified; NSCLC, non-small-cell lung carcinoma; ORR, overall response rate; OS, overall survival; OSCC, oral squamous cell carcinoma; PDAC, pancreatic ductal adenocarcinoma; PDX, patient-derived xenograft; PFS, progression-free survival; PI3K, phosphoinositide 3-kinase; PLCγ2, phospholipase C gamma 2; p-SYK, phosphorylated SYK; PTCL, peripheral T-cell lymphoma; qPCR, quantitative polymerase chain reaction; scRNA-seq, single-cell RNA sequencing; SLL, small lymphocytic lymphoma; STAT3, signal transducer and activator of transcription 3; SYK, spleen tyrosine kinase; TAM, tumor-associated macrophage; TCGA, The Cancer Genome Atlas; TCR, T-cell receptor; TFH, T-follicular helper; TIL, tumor-infiltrating lymphocyte; TNBC, triple-negative breast cancer; TNF, tumor necrosis factor.
