# Supplementary material for: Spleen Tyrosine Kinase in Malignancies and Sepsis: Pathophysiological Roles and Biomarker Potential—A Narrative Review
Source: Biomolecules. 2026 Oct 20;16(10):1529. doi: 10.3390/biom16101529 (PMC13702482; doi:10.3390/biom16101529)
Supplement: Supplementary file 1 [file biomolecules-16-01529-s001.zip › Supplementary_Table_S2.pdf]

Table S2. Experimental and Clinical Evidence Supporting the Involvement of SYK in Sepsis

Study design/model	Evidence level/type	Sample size	Main SYK-related finding	Main biological/clinical relevance	Ref.
Murine sepsis-associated AKI; neutrophils and dendritic cells	Preclinical — murine	N/A	Increased total SYK expression; R406 reduced neutrophil oxidative markers and dendritic-cell cytokines.	Renal protection after pharmacological intervention; expression is not an activation assay, and exclusive target attribution is unconfirmed.	[8]
Human endothelial cells; endothelial SYK depletion/kinase-defective construct; murine CLP	Mechanistic cellular + preclinical murine	N/A	SYK function supported VE-cadherin Tyr658/Tyr685 phosphorylation; genetic and pharmacological perturbation preserved the barrier.	Convergent evidence for endothelial SYK in permeability and lung injury; survival benefit is murine.	[9]
Human whole-blood transcriptomic reanalysis; murine CLP with post-insult PRT062607	Mixed observational human transcriptomic + preclinical murine	Human dataset: 81 postoperative septic-shock patients + 15 healthy volunteers; mice: 30 allocated, 10/group; endpoint n varies.	Increased human SYK transcript abundance; increased murine cardiac p-SYK; PRT062607 reduced p-SYK and organ-injury endpoints.	Human abundance association and murine pharmacological target engagement; no patient therapeutic efficacy test.	[10]
K. pneumoniae septic-shock challenge; serial WBN/LDN phenotyping and tissue staining	Translational — nonhuman primate	6 infected primates; 2 allocated to fostamatinib in a concurrent blinded evaluation.	Time-dependent total SYK abundance/SYK-positive populations associated with neutrophil features, renal and coagulation indices.	Cellular and temporal association with organ dysfunction; no unblinded treatment-efficacy comparison.	[11]
Sepsis blood phenotyping, severity associations, patient-neutrophil ex vivo LPS/R406	Clinical observational + human ex vivo	35 patients + 18 healthy donors; clustering: 23 patients; ex vivo stimulation/inhibition: 16 patients.	Total SYK and SYK-positive WBN/LDN populations associated with severity; R406 altered phenotypes and reduced LDN MPO release ex vivo.	Candidate cell-specific severity marker; conventional SYK staining did not assess p-SYK; no clinical intervention.	[12]
Macrophage receptor experiments; murine endotoxemia and septic peritonitis	Mechanistic cellular + preclinical murine	N/A	CD300b–TLR4/adaptor cooperation recruited SYK–PI3K and altered MyD88/TRIF outputs.	Genetic/receptor-transfer experiments support receptor integration in experimental shock; not clinical biomarker validation.	[59]
Healthy-donor neutrophils stimulated ex vivo with bacterial LPS ± R406	Human ex vivo — mechanistic pharmacological	Endpoint-specific: 3–6 donors/experiments; unique total donor count NR.	R406 reduced NETs, degranulation, ROS, adhesion, and CD16low phenotypes; selected measured functions were retained.	Laboratory pharmacological response; neither clinical sepsis efficacy nor complete antimicrobial preservation established.	[61]

Study design/model	Evidence level/type	Sample size	Main SYK-related finding	Main biological/clinical relevance	Ref.
Human monocyte perturbation; CD16-positive monocytes from patients with sepsis	Mixed mechanistic human cellular + patient-derived observational/ex vivo	Patient and healthy-donor cohort sizes NR.	CD16–SYK signaling modified TRIF/TLR4 responses; corresponding gene-expression patterns occurred in patient-derived subsets.	Supports receptor-dependent immune reprogramming; does not demonstrate a SYK-guided clinical intervention.	[62]

Note: Representative original studies are shown; evidence level/type is identified explicitly. Sample sizes for human and nonhuman primate studies are reported when verifiable from the original publication; distinct cohorts or endpoint subsets are not summed. N/A, patient/participant sample size not applicable to the cellular or murine model component; NR, not reported or not reliably retrievable. The murine allocation is included for Verra et al. because the mixed design is otherwise easily misinterpreted; model sample sizes are not intended as an exhaustive extraction. Reference numbers correspond to the revised main manuscript. Abbreviations: AKI, acute kidney injury; CD, cluster of differentiation; CLP, cecal ligation and puncture; IKK, inhibitor of nuclear factor kappa B kinase; LDN, low-density neutrophil; LPS, lipopolysaccharide; MPO, myeloperoxidase; MyD88, myeloid differentiation primary response protein 88; NET, neutrophil extracellular trap; PI3K, phosphoinositide 3-kinase; p-SYK, phosphorylated spleen tyrosine kinase; ROS, reactive oxygen species; SYK, spleen tyrosine kinase; TLR, Toll-like receptor; TRIF, TIR-domain-containing adaptor-inducing interferon beta; WBN, whole-blood neutrophil.
