# Supplementary material for: Spleen Tyrosine Kinase in Malignancies and Sepsis: Pathophysiological Roles and Biomarker Potential—A Narrative Review
Source: Biomolecules. 2026 Oct 20;16(10):1529. doi: 10.3390/biom16101529 (PMC13702482; doi:10.3390/biom16101529)
Supplement: Supplementary file 1 [file biomolecules-16-01529-s001.zip › Supplementary_Table_S3.pdf]

Table S3. Comparative Framework of SYK Biology in Malignancies and Sepsis

Dimension	Malignancies	Sepsis	Comparative/interpretative significance	Ref.
Predominant compartment	Malignant B/myeloid cells; epithelial tumor cells and infiltrating immune cells.	Neutrophils, monocytes/macrophages, endothelium; mixed circulating/tissue populations.	Identify the cellular source before assigning pathway or biomarker meaning.	[4–6, 9, 11, 12]
Upstream architecture	BCR scaffolds; oncogenic cooperation or fusion; tumor/microenvironmental inputs.	Fc receptors, CLRs, integrins; adaptor-mediated cooperation with TLR4.	Shared kinase, distinct receptor platforms.	[2, 26, 27, 39, 40, 55, 58, 59]
Activation pattern	Tonic/chronic active signaling in selected neoplasms; heterogeneous solid-tumor states.	Stimulus-driven responses that vary by receptor and population.	Sustained dependence is not equivalent to acute immune responsiveness.	[26, 27, 55, 59]
Temporal behavior	Persistent circuits with modulation by microenvironmental stimuli.	Changing stimulation, cell populations, and immune programs; early severity-associated transcriptomic endotypes.	Abundance trajectories and phosphorylation kinetics require separate assessment.	[11, 29, 67–69]
Shared modules	PI3K–AKT, MAPK, NF-κB; B-cell BLNK–BTK–PLCγ2 scaffold.	PI3K–AKT, MAPK, NF-κB; CLR–CARD9–BCL10–MALT1 coupling.	Downstream overlap does not imply identical output.	[21, 22, 26, 27, 55, 59]
Biological outputs	Survival and metabolic vulnerability, invasion, or tumor restraint; immune modulation. AML replacement evidence is pharmacological/metabolic.	Host defense, cytokines, oxidative responses, barrier injury, immune reprogramming.	The direction of benefit from inhibition differs by compartment.	[8, 9, 32, 33, 35, 45, 50, 61, 62]
Disease-specific functions	ITK–SYK fusion; isoform-dependent effects; DNA-repair resistance.	Experimental VE-cadherin disruption and acute organ injury.	No direct equivalence between these mechanisms.	[9, 10, 39, 40, 49, 51]
Total SYK expression	May reflect tumor lineage, isoform balance, or immune infiltration.	May reflect cellular abundance, composition, or severity-associated neutrophil subsets.	Expression is not proof of kinase activation.	[11, 12, 32, 33, 49, 53, 54]
p-SYK interpretation	Site-defined regulatory signal or activity surrogate; cellular/model validation needed.	Potential dynamic signaling readout; human neutrophil abundance studies are not p-SYK studies.	Specify site, assay, source, timing, and functional support.	[10, 12, 14, 38]
Biomarker maturity	Human tissue/outcome studies; candidate ex vivo response signatures.	Limited human severity associations and ex vivo observations.	Oncology is further developed, but predictive clinical validation remains incomplete.	[12, 38, 43, 49, 53, 79]
Therapeutic rationale	Disrupt malignant dependency or modify tumor immunity; early trials include dual SYK/FLT3 mivavotinib and entospletinib–obinutuzumab combination therapy.	Reduce injurious host responses while preserving pathogen control; experimental protection.	Clinical antitumor activity does not establish efficacy in sepsis.	[8–10, 31, 61, 70–72]

Dimension	Malignancies	Sepsis	Comparative/interpretative significance	Ref.
Translational constraint	Tumor/compartment heterogeneity; dual-target and combination pharmacology, toxicity, and incomplete biomarker selection.	Model heterogeneity, timing, host-defense trade-offs, limited clinical validation.	Interpret pharmacology and evidence level separately.	[9, 10, 12, 31–33, 53, 70, 72]

Note: AKT, protein kinase B; BCL10, B-cell lymphoma/leukemia 10; BCR, B-cell receptor; BLNK, B-cell linker protein; BTK, Bruton's tyrosine kinase; CARD9, caspase recruitment domain-containing protein 9; CLR, C-type lectin receptor; ITK, interleukin-2-inducible T-cell kinase; MAPK, mitogen-activated protein kinase; MALT1, mucosa-associated lymphoid tissue lymphoma translocation protein 1; NF-κB, nuclear factor kappa B; PI3K, phosphoinositide 3-kinase; PLCγ2, phospholipase C gamma 2; p-SYK, phosphorylated spleen tyrosine kinase; SYK, spleen tyrosine kinase; TLR4, Toll-like receptor 4; VE-cadherin, vascular endothelial cadherin.
