# Supplementary material for: Spleen Tyrosine Kinase in Malignancies and Sepsis: Pathophysiological Roles and Biomarker Potential—A Narrative Review
Source: Biomolecules. 2026 Oct 20;16(10):1529. doi: 10.3390/biom16101529 (PMC13702482; doi:10.3390/biom16101529)
Supplement: Supplementary file 1 [file biomolecules-16-01529-s001.zip › Supplementary_Table_S4.pdf]

Table S4. Analytical Approaches for SYK Assessment and Their Potential Biomarker Applications

Analytical method	SYK parameter assessed	Sample/material	Main advantages	Main limitations	Potential biomarker application	Ref.
RT-qPCR	SYK mRNA; potentially SYK transcript variants	Blood cells, isolated leukocytes, tumor tissue, cultured cells	High analytical sensitivity; relatively accessible; allows transcript-specific assays	Does not directly measure kinase activity; affected by RNA quality and cellular composition	Assessment of transcriptional expression and comparison between disease states or cellular populations	[49, 73]
Western blotting	Total SYK and specific phosphorylated SYK forms	Cell or tissue lysates	Distinguishes total from phosphorylated protein; allows assessment of selected phosphorylation sites	Requires relatively large sample quantities; semiquantitative; lacks single-cell resolution	Confirmation of SYK abundance and site-specific phosphorylation; pharmacodynamic assessment	[14]
Immunohistochemistry	Total SYK or p-SYK; subcellular localization	Formalin-fixed paraffin-embedded tissue, bone marrow biopsies	Preserves tissue architecture; permits spatial assessment and localization	Preanalytical variability; antibody-dependent; semiquantitative scoring; phospho-epitope instability	Total-SYK tissue characterization [74]; separate p-SYK prognostic investigation and phosphorylation assessment [38]	[38, 74]
Conventional flow cytometry	Intracellular total SYK in phenotypically defined cell populations	Whole blood, bone marrow, isolated leukocytes, cell suspensions	Single-cell resolution; multiparametric immunophenotyping; identifies cell-specific expression	Requires standardized fixation/permeabilization and gating; protocol-dependent	Characterization of SYK-positive malignant or immune-cell subsets	[12, 74]
Phospho-flow cytometry	Phosphorylated SYK at defined residues	Whole blood, bone marrow, isolated leukocytes, tumor-cell suspensions	Measures site-specific regulatory phosphorylation at single-cell level; combines signaling readouts with immunophenotype; compatible fluorescent cell barcoding can support multiparametric workflows	Highly sensitive to sampling time, stimulation, fixation, permeabilization, and antibody specificity	Candidate biomarker of regulatory phosphorylation; pharmacodynamic monitoring; identification of responsive subpopulations	[75]
Mass spectrometry-based phosphoproteomics	SYK-associated phosphorylation sites and networks; targeted E-cadherin/catenin analysis in the cited study	Cell or tissue lysates	Site/pathway discovery; the cited example combines mass-spectrometric phosphorylation analysis with biochemical and epithelial-model validation	High cost and technical complexity; intensive sample processing; limited routine availability; SYK dependence and direct substrate assignment require perturbation and validation	Mechanistic biomarker discovery and identification of signaling or therapeutic-response signatures	[77]

Analytical method	SYK parameter assessed	Sample/material	Main advantages	Main limitations	Potential biomarker application	Ref.
Single-cell transcriptomic approaches	Cell-specific SYK transcript expression within heterogeneous populations	Newly profiled cellular samples or existing single-cell expression datasets; cited studies reanalysed public datasets	Resolves cellular heterogeneity; identifies the cellular source of SYK expression; enables integration with broader transcriptional states	Transcript abundance does not establish SYK activation; relatively high cost and computational complexity; sparse transcript detection may occur; bulk transcriptomics and computational infiltration estimates must be distinguished from single-cell dataset reanalysis	Identification of SYK-expressing cellular subsets and integration into multidimensional molecular signatures	[33, 54]

Note: Total SYK expression and phosphorylated SYK should be interpreted as distinct analytical endpoints, because protein abundance does not necessarily reflect kinase activation. Site-specific phosphorylation is a regulatory readout whose functional interpretation requires appropriate validation. Reference numbers correspond to the revised main manuscript. Abbreviations: mRNA, messenger RNA; p-SYK, phosphorylated spleen tyrosine kinase; RT-qPCR, reverse transcription quantitative polymerase chain reaction; SYK, spleen tyrosine kinase. NanoBRET provides a complementary cellular inhibitor-target-engagement assay [76]; it does not directly measure catalytic activity or establish clinical biomarker validity.
